# Ratatosk: hybrid error correction of long reads enables accurate variant calling and assembly

**DOI:** 10.1186/s13059-020-02244-4

**Published:** 2021-01-08

**Authors:** Guillaume Holley, Doruk Beyter, Helga Ingimundardottir, Peter L. Møller, Snædis Kristmundsdottir, Hannes P. Eggertsson, Bjarni V. Halldorsson

**Affiliations:** 1 deCODE genetics/Amgen Inc., Reykjavík, Iceland; 2 Department of Biomedicine, Aarhus University, Aarhus, Denmark; 3 School of Technology, Reykjavik University, Reykjavík, Iceland

## Abstract

A major challenge to long read sequencing data is their high error rate of up to 15%. We present Ratatosk, a method to correct long reads with short read data. We demonstrate on 5 human genome trios that Ratatosk reduces the error rate of long reads 6-fold on average with a median error rate as low as 0.22 %. SNP calls in Ratatosk corrected reads are nearly 99 % accurate and indel calls accuracy is increased by up to 37 %. An assembly of Ratatosk corrected reads from an Ashkenazi individual yields a contig N50 of 45 Mbp and less misassemblies than a PacBio HiFi reads assembly.

**Supplementary Information:**

The online version contains supplementary material available at (10.1186/s13059-020-02244-4).

## Introduction

In Norse mythology, the squirrel Ratatöskr runs up and down the ash tree Yggdrasil, bearing envious words between the eagle at the top and the dragon at the bottom. Short read sequencing (SRS) has allowed for the accurate identification of small variants (SNPs and indels) in non-repetitive parts of the genome while long read sequencing (LRS) allows for the characterization of large and complex variations. We have designed Ratatosk to carry information between the two technologies with the hope of leveraging the benefits of both of them.

Oxford Nanopore Technologies (ONT) and Pacific Bioscience (PacBio) are LRS platforms [1] that produce long sequence reads ranging from 10^3^ to 10^6^ bases with an error rate up to 15% [2]. The high error rate of LRS reads is in part compensated by their lengths which increase their mapping accuracy, making LRS suitable for numerous applications in all fields of genomics. LRS used at high coverage on a few individuals [3] or low-medium coverage at population scale [4] greatly improves the detection of structural variants (SVs) because the large size of ONT reads spans SV breakpoints. Additionally, LRS reads can encompass large sections of highly repetitive regions in the human genome such as centromeres [5], telomeres [6], and tandem repeats [7]. Analyzing these regions with SRS is grueling as the reads generally map ambiguously to multiple locations because of their limited size. Yet, centromeres play an important role in cancer genomics [8] while short tandem repeat (STR) expansions associate with a number of genetic diseases [9]. LRS technologies have also enabled de novo haplotype-resolved assemblies with very few contig breaks [10, 11]. Finally, LRS technologies overcome chemistry limitations of SRS, in particular GC bias [12] and PCR amplification artifacts [13] causing uneven coverages for reads produced by Illumina platforms. Yet, the high error rate of LRS reads introduces algorithmic challenges in analyzing these data while filtering out the noise [14]. Highly accurate LRS technologies [15] that perform circular sequencing and generate highly accurate consensus sequences are emerging but the required resources are still prohibitive at a population scale. SRS data are therefore often used to complement to LRS data for SV breakpoint refinement [16] and assembly polishing [17].

We present Ratatosk, a new method based on a compacted and colored de Bruijn graph for the hybrid correction of genomic LRS reads using SRS data. Ratatosk is specifically designed to avoid over-correction with incorrect haplotypes or homologous regions as this would either remove true variants or add artificial ones. Ratatosk introduces several new features not included in other hybrid correction tools. First, SRS and LRS reads color vertices of the de Bruijn graph to highlight existing paths for the correction. Graph coloring enables pruning the search space when traversing the graph by removing chimeric paths. Second, LRS reads are anchored to the graph using both exact and inexact *k*-mer matches. The latter improves the anchoring of highly erroneous regions of the LRS reads. Third, the graph is annotated with candidate SNPs to disentangle small variations between haplotypes that are difficult to capture from erroneous LRS reads. Fourth, two passes of correction are performed using SRS and LRS reads separately to take advantage of all data available, as well as increasing *k*-mer sizes to remove errors made during the first correction pass. Finally, an optional reference-guided preprocessing of the input data is proposed to improve the error rate and scale Ratatosk to a large number of compute nodes.

The performance of LRS read error correction tools is usually evaluated by the error rate, genome coverage, and different assembly metrics of the corrected reads [18, 19]. However, the characterization of variants from the corrected data has yet to be investigated. Additionally, it is often unclear whether hybrid error correction tools scale to large input data as they are usually evaluated on small non-human genomes such as yeast or bacteria. In this paper, we demonstrate that Ratatosk can reduce the raw error rate of long reads 6-fold on average with a median error rate as low as 0.22 % on 5 human genome trios. Ratatosk corrected data maintain nearly 99 % accurate SNP calls and substantially increase indel calls accuracy by up to 37 % compared to the raw data. An assembly of the Ashkenazi individual HG002 [20] created from Ratatosk corrected ONT reads yields a contig N50 of 45 Mbp and less misassemblies than an assembly created from PacBio HiFi reads.

### Previous work

Methods for correcting genomic LRS reads belong to one of two categories: self-correction or hybrid correction. Self-correction methods refine the reads using information from the set of LRS reads alone while hybrid correction methods use information from a set of SRS reads originating from the same individuals. Overall, hybrid correction methods have been shown to outperform self-correction methods in terms of error rate and compute resource usage [21]. However, a recurrent issue with most error correction methods is that they do not retain the phasing of the reads, hence limiting the usage of corrected data to mixed-haplotype assembly. We provide here a short overview of hybrid correction methods and refer to genomic [19, 21, 22] and transcriptomic [23] LRS reads correction reviews for more details about self-correction methods.

LoRDEC [24] was the first method to use a de Bruijn graph built from SRS reads as an index for the correction of LRS reads. The de Bruijn graph has been extensively used as a data structure for genome assembly [25, 26] and later for SRS reads correction [27]. In LoRDEC, LRS reads are anchored on the graph using shared *k*-mers and non-anchoring subsequences are then corrected using paths which are similar to the uncorrected subsequences. Many hybrid error correction tools for LRS reads, including Ratatosk, are based on the core ideas of LoRDEC. Jabba [28] is derived from the LoRDEC method besides that SRS reads are self-corrected before graph construction and LRS reads are anchored to the graph using maximum exact matches to enable different *k*-mer lengths during correction. HG-CoLoR [29] also uses self-corrected SRS reads and aligns them to the LRS reads to find overlaps. These overlaps anchor the reads onto a variable-order de Bruijn graph allowing for multiple *k*-mer lengths. Finally, FMLRC [30] indexes the de Bruijn graph using a multi-string Burrows-Wheeler Transform of the SRS reads. This representation is lightweight in memory, enables multiple *k*-mer lengths and stores implicitly *k*-mer frequencies. FMLRC has two passes of correction, one using a short *k*-mer and one using a long *k*-mer in order to simplify the graph for high complexity regions to correct. Unlike the above tools, CoLoRMap [31] constructs a weighted alignment graph from the mapping of the SRS reads to the LRS reads. The mapping provides paths in the graph that maximize the similarity with the subsequences to correct. CoLoRMap takes advantage of the paired-end information to leap over regions of LRS reads where no SRS reads map. We refer to LRS reads correction reviews [19, 21, 22] for further information.

## Results

We evaluated Ratatosk [32] using our reference-guided preprocessing on a set of 4 Icelandic trios (I1-4) from deCODE genetics [33] and one Ashkenazim trio (HO and HP) from Genome In A Bottle [20]. Ratatosk is available at https://github.com/DecodeGenetics/Ratatosk. Each trio was sequenced with both Illumina and ONT platforms in addition to the PacBio platform for the Ashkenazim trio (see the “Availability of data and materials” section). Genome coverage and N50 metrics are reported in Table 1 for the raw long reads. The short reads used are Illumina paired-end reads of length 151 bases with a mean coverage of 42x in the Icelandic trios and 61x in the Ashkenazim trio. The Ratatosk corrected reads were subsequently compared to the raw and FMLRC [30] corrected reads. FMLRC is a reference-free hybrid correction tool for long reads with one of the best overall performance among hybrid methods [21, 22]. Time and memory usage for Ratatosk and FMLRC are reported in Additional file 1. On average, FMLRC is 28 % faster than Ratatosk and uses 39 % less memory. All reads were subsequently aligned with minimap2 [34] using the default ONT or PacBio setting for further analysis. All tools requiring a reference genome used the GRCh38.p13 human genome reference.

**Table 1:** Genome coverage and N50 for the long reads of the child (C), father (F), and mother (M) in 4 Icelandic trios (I1-4) and one Ashkenazim trio (H)

	Sequencing	Coverage			N50		
	platform	C	F	M	C	F	M
I1	ONT	63.68	50.94	64.74	20,353	24,093	23,528
I2	ONT	55.68	67.95	70.46	25,767	22,496	20,439
I3	ONT	69.50	57.05	56.62	24,047	27,787	26,856
I4	ONT	57.07	57.40	64.28	23,111	15,234	26,634
HO	ONT	46.72	85.23	87.60	52,311	45,924	49,285
HP	PacBio	73.85	35.03	32.77	11,065	10,691	10,617

### Error rate

Table 2 shows the error rates for the uncorrected long reads as well as the long reads corrected by Ratatosk and FMLRC. The mean error rate of the Ratatosk reads is about 2.16 times lower than the FMLRC reads and about 6.21 times lower than the raw reads. In the PacBio data set of the Ashkenazim trio, 50 % of the Ratatosk reads have an error rate of 0.34 % or below. This is up to 42.58 times lower than the raw reads and up to 15.02 times lower than the FMLRC reads. Details on the error rate calculations are given in Additional file 1.

**Table 2:** Error rates (in %) for the raw and corrected long reads in 4 Icelandic trios and one Ashkenazim trio. Best results are highlighted

		Raw			FMLRC			Ratatosk		
		C	F	M	C	F	M	C	F	M
Mean	I1	11.89	11.19	10.89	3.85	3.55	3.32	**1.67**	**1.73**	**1.63**
	I2	10.52	11.20	10.14	3.20	3.48	2.94	**1.62**	**1.71**	**1.49**
	I3	9.98	10.52	10.78	3.07	3.16	3.47	**1.65**	**1.58**	**1.02**
	I4	10.74	11.18	10.17	3.26	3.57	2.93	**1.47**	**1.66**	**1.44**
	HO	8.81	7.82	8.24	2.53	2.23	2.23	**1.40**	**1.37**	**1.29**
	HP	14.66	14.80	15.02	7.38	7.54	7.60	**3.03**	**3.12**	**3.08**
Median	I1	9.95	9.10	8.84	1.41	1.18	1.11	**0.24**	**0.25**	**0.23**
	I2	8.37	9.05	8.22	1.00	1.15	0.88	**0.23**	**0.24**	**0.22**
	I3	7.95	8.42	8.72	1.03	0.99	1.27	**0.27**	**0.23**	**0.27**
	I4	8.91	9.34	8.16	1.23	1.33	0.96	**0.22**	**0.22**	**0.22**
	HO	6.95	5.95	6.62	0.55	0.50	0.56	**0.27**	**0.26**	**0.26**
	HP	14.02	14.15	14.48	4.86	4.98	5.11	**0.34**	**0.34**	**0.34**

**Fig. 1:**
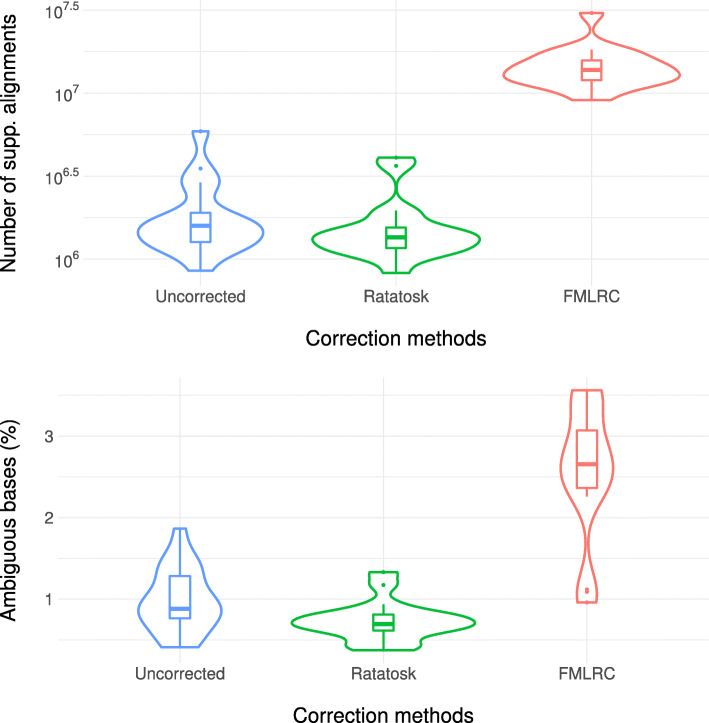
Number of supplementary alignments and ratio of ambiguous bases for the Icelandic trios and the HG002 data sets

We also show in Table 3 the ratio of aligned reads in the raw and corrected data sets. On average, the ratio of aligned reads corrected by Ratatosk is similar to the raw reads while FMLRC has 5.98 % more aligned reads compared to the raw data sets. To explain such a difference, we measured over-correction in the corrected long reads by reporting in Fig. 1 the number of supplementary alignments and the ratio of ambiguous bases. Supplementary alignments occur when an alignment cannot be represented as a single linear alignment [35] but instead, as a set of linear alignments. The presence of supplementary alignments might indicate an SV large enough for the aligner to abandon mapping the read with a single linear alignment. Supplementary alignments might also indicate that the read has been partially over-corrected. Finally, ambiguous bases are bases from reads which do not align in the extremities of primary alignments (*soft-clipping*) but do align in at least one distant supplementary alignment of the same reads. The ratio of ambiguous bases measures the proportion of read bases mapping ambiguously because of chimeric reads [36] or over-correction. More details are given in Additional file 1.

**Table 3:** Ratio of aligned reads (in %) with respect to the number of raw long reads in 4 Icelandic trios and one Ashkenazim trio. Best results are highlighted

	Raw			FMLRC			Ratatosk		
	C	F	M	C	F	M	C	F	M
I1	92.44	91.16	92.67	**99.53**	**99.33**	**99.50**	92.67	91.39	92.89
I2	93.21	93.15	85.72	**99.54**	**99.53**	**94.46**	93.45	93.37	86.00
I3	93.98	92.59	92.75	**99.54**	**99.50**	**99.46**	94.18	92.85	86.50
I4	95.03	94.64	93.87	**99.64**	**99.59**	**99.59**	95.20	94.83	94.07
HO	42.71	60.31	46.47	**48.48**	**67.00**	**51.57**	43.02	60.72	46.79
HP	93.43	92.94	92.89	**97.27**	**97.11**	**97.02**	94.03	93.56	93.51

As shown in Fig. 1, Ratatosk decreases on average the number of supplementary alignments by 16.22 % and the ratio of ambiguous bases by 25.15 % compared to the raw reads. On the other hand, FMLRC increases the number of supplementary alignments by a factor 7.56 and increases the ratio of ambiguous bases by a factor 2.62. This suggests that Ratatosk can correct soft-clipped bases and chimeric reads while FMLRC is susceptible to over-correction. This could be partially explained by the orthogonal approach of each respective tool regarding their default *k*-mer length. On one hand, FMLRC uses short *k*-mers to increase the number of anchors at the expense of graph contiguity. On the other hand, Ratatosk uses longer *k*-mers but an inexact anchoring to maintain a good trade-off between the number of anchors and graph contiguity.

Subsampling was performed on the ONT reads of the Ashkenazim trio as reported in Additional file 1. Each raw data set was subsampled at 10x, 20x, and 30x ONT coverage. The subsampled ONT reads were thereafter corrected with Ratatosk using Illumina reads subsampled at 30x coverage. Even at 10x coverage, Ratatosk corrected reads maintain a similar error rate and ratio of aligned reads as with the full coverage data sets.

### Variant calling

There is a limited number of tools that can perform small variant calling on corrected LRS reads. Clair [37] and DeepVariant [38] are machine learning based and can train a model given a training set of input reads. We used Clair for our evaluations as DeepVariant could not be trained on raw ONT reads due to time and memory requirements. Longshot [39] was not used as it does not call indels while Medaka [40] uses an error model specific to the raw ONT reads and hence, could not be applied to corrected data. A model was trained with Clair on the raw, FMLRC, and Ratatosk ONT reads from the Ashkenazim trio using the truth set v4.2 of variants less than 50 bases long in the high confidence regions [41]. The different models generated for each type of input long reads were then used to call small variants on all genomes and variant calls were subsequently evaluated using rtg-tools [42]. Specifically, the HG003 models were used to call small variants on HG002 and HG004 while the HG002 models were used to call small variants on HG003 and the 4 Icelandic trios. While HG002 and HG003 present a risk of over-fitting as the individuals are related, we show in the following that their variant calling accuracy is similar to the one of HG004 which was called with a model trained on an unrelated individual.

Given a variant truth set, rtg-tools automatically computes an optimal quality score threshold for the variant calls. Table 4a shows the variant calls accuracy for the Ashkenazim trio for which low quality variants below the optimal threshold are filtered out (thresholds are provided in Additional file 1). On the other hand, Table 4b illustrates a standard setting for which all variants with the FILTER field set to PASS in the VCF files are used. With quality score filtering, SNP calls are nearly 99 % accurate for the raw and Ratatosk reads with a slight accuracy decrease in the SNPs called from the FMLRC reads. This demonstrates that SNPs are accurately represented in the raw reads and Ratatosk captures well the SNP candidates in the correction. However, indels are poorly represented in the raw reads and Ratatosk increases the indel calls accuracy by up to 37.56 % compared to the raw reads. When no filtering is applied, the difference of indel calls accuracy between raw and corrected reads is staggering. Indeed, the indel calls accuracy of raw reads shrinks to 20.02 % because a larger number of false positive indels are called compared to the filtered calls. Indel call accuracy from the FMLRC reads decreases to 73.23 % while indels called from the Ratatosk reads decline only to 90.80 % accuracy. Variant calling performed on the subsampled data sets in Additional file 1, indicates that only as low as 20x ONT and 30x Illumina coverages are required to maintain similar performance as with full coverage.

**Table 4:** Small variant calls accuracy (in %) for the ONT reads from the Ashkenazim trio in the high confidence regions. Best results are highlighted

		SNPs			Indels		
		Precision	Recall	F1	Precision	Recall	F1
(a) Variants with quality scores below a threshold automatically computed by rtg-tools are filtered out							
HG002	Raw	**98.87**	97.93	98.25	81.83	39.26	53.06
	FMLRC	96.42	96.31	96.37	89.96	80.55	85.00
	Ratatosk	97.84	**99.10**	**98.47**	**92.10**	**89.19**	**90.62**
HG003	Raw	**99.16**	98.85	99.00	84.00	47.62	60.78
	FMLRC	97.73	97.56	97.64	92.78	85.38	88.93
	Ratatosk	98.94	**99.42**	**99.18**	**94.04**	**92.14**	**93.08**
HG004	Raw	**99.19**	98.73	98.96	83.67	45.50	58.94
	FMLRC	97.22	97.44	97.33	90.11	84.52	87.23
	Ratatosk	98.56	**99.46**	**99.01**	**92.65**	**91.38**	**92.01**
(b) All variants with the FILTERfield set to PASS in the VCF files are used							
HG002	Raw	85.32	99.69	91.94	11.60	72.96	20.02
	FMLRC	78.08	99.57	87.52	60.20	93.47	73.23
	Ratatosk	**90.22**	**99.82**	**94.78**	**86.07**	**96.09**	**90.80**
HG003	Raw	95.15	99.74	97.39	15.44	77.19	25.73
	FMLRC	86.73	99.58	92.72	69.99	93.26	79.97
	Ratatosk	**95.94**	**99.80**	**97.83**	**88.38**	**95.83**	**91.95**
HG004	Raw	**93.34**	99.80	**96.47**	14.83	75.98	24.81
	FMLRC	84.08	99.68	91.21	66.87	94.60	78.35
	Ratatosk	93.05	**99.87**	96.34	**87.77**	**96.69**	**92.02**

No variant truth set is available for the Icelandic trios so Mendelian inheritance concordance was measured by rtg-tools instead, as shown in Table 5. Overall, small variants calls from Ratatosk reads are the most consistent with the calls from each parents and both parents across most trios.

**Table 5:** Mendelian concordance (in %) of small variants called on the ONT reads of 4 children from Icelandic trios with respect to the variant calls from their father (F), mother (M), and both parents (F+M). All variants with the FILTER field set to PASS in the VCF files are used by rtg-tools. Best results are highlighted

	Raw			FMLRC			Ratatosk		
	F	M	F+M	F	M	F+M	F	M	F+M
I1	99.24	99.28	95.86	99.18	99.21	97.11	**99.42**	**99.42**	**98.06**
I2	99.28	99.22	96.22	99.24	99.22	97.33	**99.45**	**99.44**	**97.82**
I3	**99.31**	**99.33**	96.14	99.05	99.13	**96.91**	98.86	98.25	96.05
I4	99.19	99.38	96.76	99.19	99.33	97.33	**99.46**	**99.50**	**98.28**

### Assembly

The raw and Ratatosk corrected ONT reads of HG002 were assembled using Flye 2.8.1 [17]. We compared the Flye assemblies to a recent assembly made from PacBio HiFi reads with HiCanu [43, 44] and the reference assembly Ash1 v1.7 [45, 46] made from Illumina, ONT, and PacBio HiFi reads assembled with MaSuRCA [47]. The Flye and HiCanu assemblies were post-process with purge_dups [48] to exclude allelic contigs from the assemblies. All assemblies were evaluated with QUAST 5.0.2 [49] and Merqury [50]. Misassemblies reported by QUAST were filtered to exclude errors in known SVs [51] and segmental duplication sites as well as centromeric, telomeric, and gap regions using a script from HELEN [52]. The quality value represents a log-scaled probability of error for the consensus basecalls while the *k*-mer completeness measures the proportion of *k*-mers shared between the assembly and an accurate SRS data set from the same individual.

As shown in Table 6, the Flye assembly of the Ratatosk reads is competitive with other high quality LRS assemblies. In particular, the Ratatosk/Flye assembly displays a similar *k*-mer completeness, contig N50, number of contigs and number of misassemblies as the HiFi/HiCanu assembly. However, the Ratatosk/Flye assembly has the largest NA50 and the lowest rates of mismatches and indels while the HiFi/HiCanu assembly shows the best quality value due to the high accuracy of HiFi reads. While all assemblies have a similar *k*-mer completeness, the Ash1 reference assembly has the best reference genome GRCh38 coverage. However, 1.96 % of the Ash1 assembly is derived from the reference genome GRCh38. Overall, these results demonstrate that the correction performed by Ratatosk is suited for producing highly contiguous assemblies of quality with very few errors. A natural extension of this work is haplotype-aware assembly [53] and variant calling from highly contiguous haplotigs [54].

**Table 6:** HG002 assembly statistics for the Flye and HiCanu assemblies as well as the Ash1 reference assembly. Misassemblies are filtered to exclude errors in known SVs and segmental duplication sites as well as centromeric, telomeric, and gap regions. All metrics are computed by QUAST except *k*-mer completeness and quality value which are computed by Merqury. Best results are highlighted

	ONT Flye	ONT+Ratatosk Flye	PacBio HiFi HiCanu	Ash1
Reference coverage (%)	94.90	95.85	96.71	**98.50**
*k*-mer completeness (%)	95.79	97.27	97.45	**97.67**
Quality value	33.97	47.90	**55.17**	41.341
N50 (Mbp)	37.98	**45.05**	44.67	34.30
NA50 (Mbp)	20.83	**25.46**	19.78	16.48
# contigs	972	430	**422**	2,412
# misassemblies	**68**	75	84	188
# mismatches / 100 kbp	121.71	**112.66**	178.36	161.09
# indels / 100 kbp	109.67	**26.66**	26.84	27.00

## Conclusion

We present Ratatosk, a hybrid error correction tool for noisy genomic long reads designed for accurate variant calling and assembly. Ratatosk uses short and long reads to color paths in a compacted de Bruijn graph in order to highlight existing paths for the correction. The graph is also annotated with candidate SNPs to disentangle small variations between haplotypes. An inexact anchoring procedure is employed to improve the correction in highly erroneous regions of the long reads. Finally, an optional reference-guided preprocessing of the input data is proposed to improve the error rate and scale Ratatosk to a large number of compute nodes. We demonstrate on 5 human genome trios that Ratatosk decreases the error rate 6-fold on average compared to the raw reads with a median error rate as low as 0.22 %. SNPs calls on Ratatosk corrected reads are nearly 99 % accurate and indel calls accuracy is up to 37 % higher compared to the raw reads. Furthermore, variants calls obtained from 4 corrected trios are highly concordant. Finally, we show that Ratatosk corrected data enable highly contiguous assemblies with fewer errors compared to other assemblies made from accurate long reads. Future work includes running time improvements, phasing and population based correction.

## Methods

The “Definitions” section details the concepts and data structures that will be used throughout this paper. The “Graph construction and preprocessing” section describes how the main index is built and preprocessed for correction. The “First correction pass” and “Second correction pass” sections overview the methods used during the first and second correction passes, respectively.

### Definitions

**Fig. 2:**
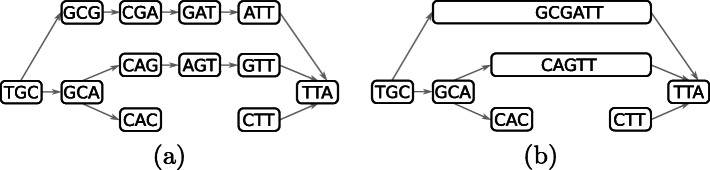
A de Bruijn graph in **a** and its compacted counterpart in **b** using 3-mers. For simplicity, the de Bruijn graph is directed and reverse-complements are not considered

A string *s* is a sequence of symbols drawn from an alphabet $\mathcal {A}$. The length of *s* is denoted by |*s*|. A substring of *s* is a string in *s* with a start position *i*, a length *l* and is denoted by *s*(*i*,*l*). Let $\mathcal {A}$ be the DNA alphabet $\mathcal {A} = \{A, C, G, T\}$ for which (*A*,*T*) and (*C*,*G*) are complementing pairs. The reverse-complemented string $\overline {s}$ is the reverse sequence of complemented symbols in *s*. The canonical string $\hat {s}$ is the lexicographically smallest of *s* and its reverse-complement $\overline {s}$. A de Bruijn graph (dBG) is a bi-directed graph *G*=(*V*,*E*) in which each vertex *v*∈*V* represents a *k*-mer and its reverse-complement. Only the canonical *k*-mer of each vertex is stored in *G*. A directed edge *e*∈*E* from vertex *v* to vertex *v*^′^ representing *k*-mers *x* and *x*^′^, respectively, exists if and only if *x*(2,*k*−1)=*x*^′^(1,*k*−1). Each edge *e* is labeled with the orientation of the *k*-mers *x* and *x*^′^ they connect: $\left \{x, x'\right \}, \left \{x, \overline {x'}\right \}, \left \{\overline {x}, x'\right \}$ or $\left \{\overline {x}, \overline {x'}\right \}$. Each *k*-mer *x* has $|\mathcal {A}|$ possible successors *x*(2,*k*−1)⊙*a* and $|\mathcal {A}|$ possible predecessors *a*⊙*x*(1,*k*−1) in *G* with $a\in \mathcal {A}$ and ⊙ as the concatenation operator. The number of *k*-mers in *G* is denoted |*G*|. A path in the graph is a sequence of connected vertices *P*=(*v*_1_,…,*v*_*m*_). Path *P* is said to be *non-branching* if it is composed of vertices having an in- and out-degree of one with exception of the head vertex *v*_1_ which can have more than one incoming edge and the tail vertex *v*_*m*_ which can have more than one outgoing edge. A non-branching path is maximal if it cannot be extended in the graph without branching. A compacted de Bruijn graph (cdBG) merges all maximal non-branching paths *P* from the dBG into single vertices, called *unitigs*, representing substrings of length |*P*|+*k*−1. A simplified dBG and its compacted representation are illustrated in Fig. 2a and b. A colored de Bruijn graph is a graph *G*=(*V*,*E*,*C*) in which (*V*,*E*) is a dBG and *C* is a set of colors such that each vertex *v*∈*V* maps to a subset of *C*. We extend the definition of a cdBG to a compacted and colored de Bruijn Graph (ccdBG) where (*V*,*E*) is a cDBG, so the vertices represent unitigs, and each *k*-mer of a unitig maps to a subset of *C*.

### Graph construction and preprocessing

**Fig. 3:**
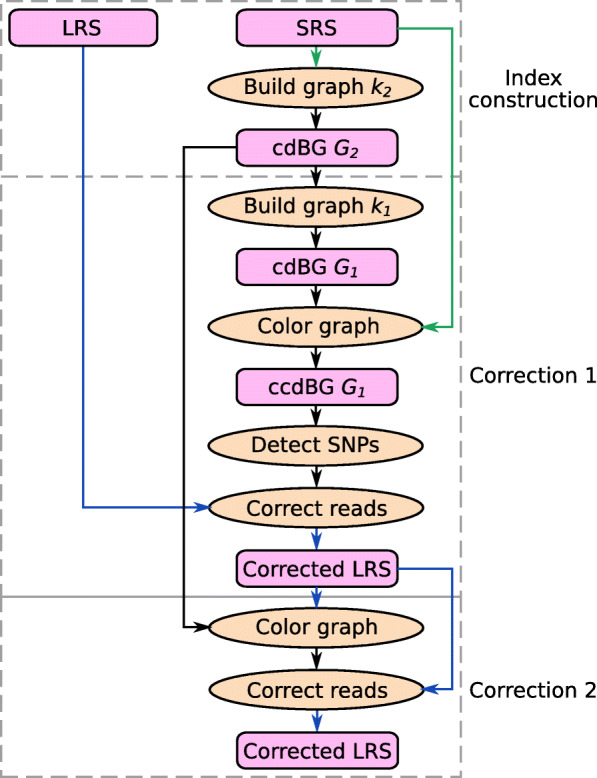
Ratatosk performs two passes of correction, each using a different *k*-mer size for the graph construction and a different type of reads for the graph coloring. LRS reads are shown in blue and SRS reads in green

Ratatosk takes as input a set $\mathcal {S}$ of paired SRS reads and a set $\mathcal {L}$ of LRS reads. A cdBG is built from $\mathcal {S}$ to correct the reads in $\mathcal {L}$ using two correction passes as shown in Fig. 3.

#### Graph construction

Using different *k*-mer lengths in the graph built from $\mathcal {S}$ has been shown to improve the correction of $\mathcal {L}$ [28–30]: A short *k*-mer is ideal for finding matches between LRS reads and the graph while unitigs built with long *k*-mers have a better contiguity. In order to combine the advantages of short and long *k*-mers, Ratatosk uses two *k*-mer lengths *k*_1_ and *k*_2_ with *k*_2_≥2*k*_1_.

First, a cdBG *G*_2_ is built with the long *k*_2_-mers of $\mathcal {S}$ using the Bifrost graph engine [55]. By default, all *k*_2_-mers occurring exactly once in $\mathcal {S}$ are assumed to contain a sequencing error and are discarded from the graph construction. Subsequently, a cdBG *G*_1_ is built from the short *k*_1_-mers of the unitigs in *G*_2_. Graph *G*_1_ is used for the first correction pass while graph *G*_2_ is later used in the second correction pass (Fig. 3, the “Second correction pass” section).

#### Graph coloring

**Fig. 4:**
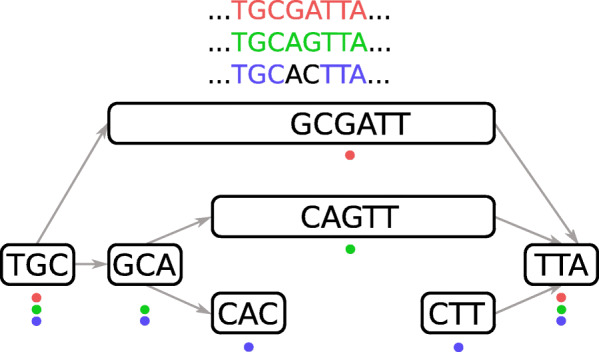
Graph coloring with three colors. Each color represents a read sharing at least one *k*-mer with a unitig

Graph *G*_1_ is turned into a ccdBG by coloring its unitigs with the read pairs from $\mathcal {S}$ with which they share at least one *k*_1_-mer, as shown in Fig. 4. Coloring unitigs with read pairs is similar to *partitions* in the guided de Bruijn graph [56] and *links* in the Linked de Bruijn graph [57]. Given $\frac {|\mathcal {S}|}{2}$ SRS read pairs in input, each pair is identified by a color identifier ranging from 1 to $\frac {|\mathcal {S}|}{2}$. Graph coloring is known to be memory consuming [55] and caution must be exercised to not overflow the memory for input high coverage data sets. For this purpose, Ratatosk enables a memory efficient graph coloring by using two techniques and a graph pruning based on *k*-mer coverage described in Additional file 1.

#### Candidate SNP annotation

While most de novo detection methods for SNPs, indels and SVs are based on the analysis of graph *bubbles* [58–61], Ratatosk uses instead a simple but fast string matching method to annotate vertices in the graph containing one or more candidate SNPs. For each *k*_1_-mer *x* in unitigs, the graph is queried for all *k*_1_-mers having a Hamming distance of 1 with *x*. Let *x*=*u*(*p*,*k*_1_) and *x*^′^=*u*^′^(*p*^′^,*k*_1_) be *k*_1_-mers from unitigs *u* and *u*^′^, respectively, that differ by exactly one substitution at position *i*<*k*_1_. Unitigs *u* and *u*^′^ are then annotated at position *p*+*i* and *p*^′^+*i*, respectively, with a IUPAC symbol representing the substitution. For example, symbol R would be assigned to position 3 in unitigs GCGATT and GCA of Fig. 4 to represent an A/G substitution.

### First correction pass

The following section describes how LRS reads are anchored to the ccdBG and the methods used to correct non-anchored regions of the LRS reads.

#### Read anchoring

We define *solid* and *weakk*-mers similarly as defined in LoRDEC and introduce the definition of *near solidk*-mers:

- Solid *k*-mer: exact length *k* substring match between a long read and a unitig from the graph.
- Near solid *k*-mer: inexact length *k* substring match between a long read and a unitig from the graph with one base substitution or indel.
- Weak *k*-mer: length *k* substring of a long read which is neither a solid *k*-mer nor a near solid *k*-mer.

We define two types of regions in a long read:

- Solid region: a region of a long read composed only of solid *k*-mers.
- Non-solid region: a region of a long read composed of weak or near solid *k*-mers.

A solid or near solid *k*-mer is also called a *match*. A match between long read *r* at position *p*_*r*_ and unitig *u* at position *p*_*u*_ is denoted *m*=〈*p*_*r*_,*r*,*p*_*u*_,*u*〉. A match *m* is *unique* if it is the only match at position *p*_*r*_ in *r*. A *k*-mer has at most one solid match in *G*_1_ but can have multiple near solid matches in *G*_1_. Note that solid and non-solid regions can overlap by *k*−1 bases. All non-solid regions are surrounded by two solid regions with the exception of non-solid regions at the start and end of LRS reads.

#### Delimiting non-solid regions

Each read $r \in \mathcal {L}$ is corrected independently, allowing multiple threads to correct LRS reads in parallel. The graph is queried for each *k*_1_-mer of *r*, resulting in a list of solid matches *M*_*s*_ and a list of near solid matches *M*_*n*_, both sorted by ascending match position *p*_*r*_ in *r*. Only unique near solid matches (UNSM) are kept in *M*_*n*_ to prevent anchoring *r* on a SNP or indel from an incorrect allele. Furthermore, a *k*_1_-mer which is both a solid match and a near solid match is considered solid and its near solid matches are discarded from *M*_*n*_.

**Fig. 5:**
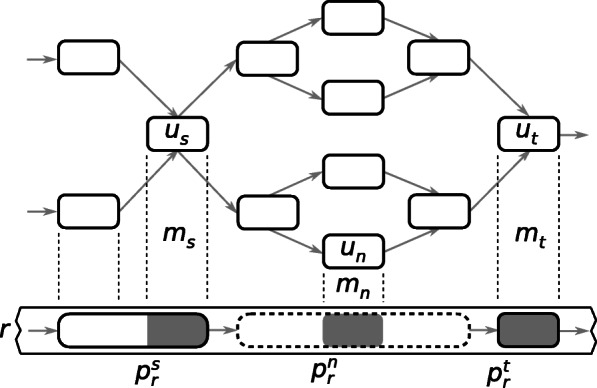
Example of a long read *r* anchored on a ccdBG. A section of *r* is shown at the bottom with two solid regions (non-dashed boxes at the extremities) surrounding a non-solid region (dashed line box at the center). The grey areas of the solid regions show the source and target matches between the long read and the graph. The grey area of the non-solid region shows a near solid match. For simplicity, colors are not shown

Non-solid regions of *r* are detected by finding all pairs of successive solid matches *m*_*s*_,*m*_*t*_∈*M*_*s*_ for which $p_{r}^{s} \neq p_{r}^{t} - 1$ with the exception of non-solid regions at the extremities of *r*. The first match *m*_*s*_ of the pair is referred to as the *source* match and the second match *m*_*t*_ of the pair is referred to as the *target* match. The length of the non-solid region to correct is then $l = p_{r}^{t} - p_{r}^{s} + k_{1}$. It includes $r\left (p_{r}^{s}, k_{1}\right)$ which is the last solid *k*_1_-mer from the source solid region and $r(p_{r}^{t}, k_{1})$ which is the first solid *k*_1_-mer from the target solid region as illustrated in Fig. 5. If a read starts with a non-solid region, that region has no source match and hence starts on the first position of the read. Similarly, if a read ends with a non-solid region, that region has no target match and hence ends on the last position of the read.

#### Traversing the graph

**Fig. 6:**
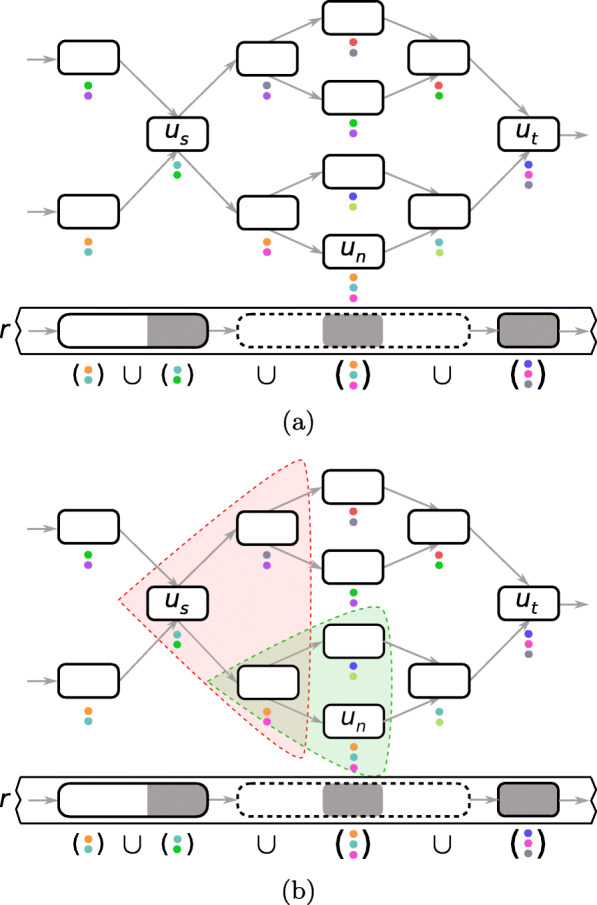
In **a**, the union of colors is computed within the solid regions around the non-solid region to correct and the USNMs. This union will partially guide the graph traversal, along with the sequence similarity of the paths to the non-solid region. In **b**, a first subgraph (highlighted in red) of all paths starting at unitig *u*_*s*_ with *P*_*max*_=2 unitigs is explored for correction. The lower path is extended using the same method (shown in green) and a path connecting to *u*_*n*_ is found

In order to correct a non-solid region, Ratatosk attempts to extract one path in the graph connecting unitig *u*_*s*_ of the source match to unitig *u*_*t*_ of the target match. Since the length *l* of the non-solid region to correct is known, we assume that the corrected path between *u*_*s*_ and *u*_*t*_ has minimum sequence length $l_{min} = \frac {l}{1 + F}$ bases and maximum sequence length *l*_*max*_=*l*·(1+*F*) bases where *F* is an upper-bound of the error rate in the long read (see Additional file 1). Ratatosk uses two greedy techniques to guide the traversal in the graph and prune the search space, as shown in Fig. 6.

First, rather than exploring all paths between unitigs *u*_*s*_ and *u*_*t*_, Ratatosk only explores paths traversing UNSMs in the non-solid region to correct. These matches provide an anchoring in the non-solid regions as they are near exact *k*_1_-mer matches between the graph and the read to correct. Hence, paths between *u*_*s*_ and *u*_*t*_ which do not traverse the UNSMs are pruned because they are not good candidates for the correction. Let *m*_*n*_ be the near solid match from *M*_*n*_ with the smallest position $p{_{r}^{n}}$ such that $p_{r}^{s} + k_{1} \leq p{_{r}^{n}} \leq p_{r}^{t} - k_{1}$. Ratatosk first attempts to extract one path connecting unitig *u*_*s*_ to unitig *u*_*n*_∈*m*_*n*_ with a BFS traversal that only explores paths with maximum sequence length $\left (p{_{r}^{n}} - p_{r}^{s} + k_{1}\right) \cdot (1 + F)$ bases. The extracted path is then extended from *u*_*n*_ to the next UNSM in *M*_*n*_. The process of extending the last unitig of a path to the next UNSM in *M*_*n*_ is repeated until there are no more UNSMs to consider in *M*_*n*_ or no path extension is possible. Finally, the graph traversal attempts to extend the path to the target unitig *u*_*t*_. Note that in the absence of UNSM in the non-solid region to correct, all paths connecting *u*_*s*_ and *u*_*t*_ with minimum sequence length *l*_*min*_ and maximum sequence length *l*_*max*_ are traversed.

Second, even using UNSMs to prune the search space during traversal, the subgraph between two unitigs *u*_*n*_ and *u*_*n*_^′^ from UNSMs can be very large. This is particularly true for LRS reads with a high error rate, resulting in long non-solid regions with few or no UNSMs. In order to prune the search space between *u*_*n*_ and *u*_*n*_^′^, a greedy graph traversal is used to extract one path connecting the two unitigs. Unitig *u*_*n*_ is first extended by visiting all paths of length *P*_*max*_ vertices with a BFS traversal. Each traversed path is given a probability *s*_*P*_ of being the correct path to extend and only the path with the greatest probability is extended. The path chosen for extension maximizes its sequence similarity with the non-solid region to correct. Furthermore, as colors highlight paths in the graph representing SRS reads, the path chosen for extension also maximizes its color similarity with the surrounding solid regions. Hence, before correcting a non-solid region, Ratatosk first computes the union *C* of all colors sets *C*_*u*_ from the solid matches and UNSMs within an interval corresponding to the non-solid region start and end positions extended of *B* bases on each side, i.e.,

1 $$ C = \bigcup\limits_{u \in m} C_{u} \text{,} \forall m \in M_{s}, M_{n} \textrm{ with}\,\, p_{r}^{s} - B \leq p_{r} \leq p_{r}^{t} + B   $$

During the BFS traversal, a path probability *s*_*P*_ is computed for each traversed path based on the number of colors the path shares with *C* and the sequence similarity of the path to the region to correct. Specifically, given a path *P* composed of *P*_*max*_ unitigs and its color set $C_{p} = \underset {u \in P}{\cup } C_{u}$, the color matching probability of *P* is $s_{c} = \frac {|C_{p} \cap C|}{|C|}$ and the sequence matching probability *s*_*q*_ is derived from the normalized edit distance of *P* to the non-solid region to correct using an infix alignment computed by the edlib tool [62]. Both probabilities are then conflated:

2 $$ s_{P} = \frac{s_{c} \cdot s_{q}}{s_{c} \cdot s_{q} + (1 - s_{c}) \cdot (1 - s_{q})}   $$

The path with the greatest probability *s*_*P*_ is extended by starting a new graph traversal from its last unitig. The extension continues until unitig *u*_*n*_^′^ is reached or no path can be extracted as a result of a tip in the graph or extending over $\left (p{_{r}^{n'}} - p{_{r}^{n}} + k\right) \cdot (1 + F)$ bases.

To enable a faster traversal, a local minimum number of colors *T*_*C*_ is computed from the surrounding solid regions and the unitigs of UNSMs. Each traversed unitig *u* of a path *P* must be colored by at least *T*_*C*_ colors of *C* such that:

3 $$ T_{C} = D \cdot \underset{u \in m}{\min}|C_{u}| \text{,} \forall m \in M_{s}, M_{n}\,\, \text{with}\,\, p_{r}^{s} - B \leq p_{r} \leq p_{r}^{t} + B  $$

and *D* being a fixed lower bound factor (see Additional file 1). If the color set of a traversed unitig has less than *T*_*C*_ colors, its path is not explored any further nor it is considered for extension.

**Fig. 7:**
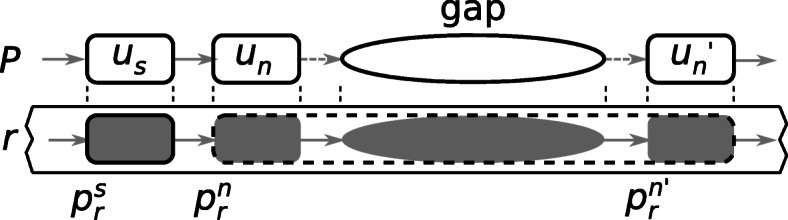
Example of a gap in a path. Path *P* is first extended until unitig *u*_*n*_, then a gap corresponding to a subsequence from the uncorrected read is inserted in *P* and the extension of *P* resumes from unitig *u*_*n*_^′^

A path extension connecting unitig *u*_*u*_ to unitig *u*_*v*_ might end prematurely for multiple reasons: all possible extensions end on a tip of the graph because of incomplete SRS data or insufficient color coverage in the traversed subgraph. In such a case, the extended path is completed with a gap corresponding to the non-solid subsequence to correct and the path extension resumes from unitig *u*_*v*_. An example of path extension with a gap is illustrated in Fig. 7.

Finally, non-solid regions located on long read extremities have only one surrounding solid region. The non-solid region at the start of a long read is corrected using a backward graph traversal from *u*_*t*_ and the one at the end of a long read is corrected with a forward graph traversal from *u*_*s*_. Because each of these graph traversals has no target match, any path with length *l* base such that *l*_*min*_≤*l*≤*l*_*max*_ is returned as a candidate for correction.

#### Forward and backward corrections

A candidate path for correction is *incomplete* if it contains a gap or if it does not connect to the unitig of a target match. If no path or only an incomplete path has been extracted, Ratatosk corrects the non-solid region backward, i.e., from the target match to the source match. Indeed, the forward graph traversal might have stopped prematurely for multiple reasons, one of which being that the color guidance led incorrectly to a tip in the graph. However, traversing the graph backward might lead to a different path. If both forward and backward paths are incomplete, Ratatosk merges both paths by aligning their sequences to the non-solid region using the Needleman–Wunsch algorithm (global alignment). The merged sequence is created by traversing the alignment of both forward and backward corrections at the same time and selecting subsequences in each corrections. In the case of candidate paths starting or ending a long read, all candidate paths are aligned to the non-solid region using a local alignment that does not penalize gaps at the end. The candidate path with the smallest edit distance is chosen for the correction.

#### Candidate SNP correction

Heuristics used to traverse the graph as presented in the “Traversing the graph” section might incorrectly extend a path and lead to the erroneous correction of a non-solid region using SNPs from incorrect alleles. Once a path has been selected to correct a non-solid region, all the positions in this path matching candidate SNPs and their IUPAC symbols are known from the unitigs. Let *s* be the non-solid region and *s*^′^ its corrected counterpart. Sequence *s*^′^ is aligned to *s* and a CIGAR string is generated from the alignment. Ratatosk iterates over matching positions of the CIGAR string (symbol M) denoted *m*=〈*s*,*p*,*s*^′^,*p*^′^〉. Note that *m* indicates that base *s*(*p*,1) is either a match or a mismatch with base *s*^′^(*p*^′^,1) but is not part of an insertion or deletion in the alignment. Let *M*_*snp*_ be the set of all matches *m*=〈*s*,*p*,*s*^′^,*p*^′^〉 for which *s*^′^(*p*^′^,1) has an assigned IUPAC symbol in the graph indicating a candidate SNP. For each match *m*=〈*s*,*p*,*s*^′^,*p*^′^∈*M*_*snp*_〉, base *b*=*s*(*p*,1) is compared to the IUPAC symbol associated to *b*^′^=*s*^′^(*p*^′^,1). If *b* is one of the possible bases represented by the IUPAC symbol, then *b*^′^ is corrected with *b*. This method enables a conservative correction of SNPs in the corrected non-solid regions by using only bases from the uncorrected non-solid regions which are compatible with the candidate SNPs from the graph. However, this method only corrects SNPs in the matching or mismatching regions of the alignment and discards candidate SNPs located within insertions of *s*^′^. To overcome this issue, a match *m*∈*M*_*snp*_ is said *strongly compatible* if *s*^′^(*p*^′^,1)=*s*(*p*,1) prior to SNP correction. A strongly compatible SNP indicates that Ratatosk is confident in the subpath that was selected to correct the region around that candidate SNP. As the strongly compatible SNP at position *p*^′^ is from unitig *u*^′^∈*m*, all bases which are candidate SNPs in *u*^′^ are used to correct SNPs in the inserted positions of the alignment (symbol I in the CIGAR string) around position *p*^′^.

### Second correction pass

In the first correction pass, Ratatosk corrected each LRS read independently from the other reads in $\mathcal {L}$. In a second correction pass, Ratatosk takes advantage of the set of corrected LRS reads as a whole. Indeed, reads corrected during the first pass might be sufficiently error-free to correct the remaining non-solid regions. Furthermore, LRS reads are at least an order of magnitude longer than SRS reads and do not need to be paired, hence offering more information to which paths to traverse in the graph. In the following, we describe the second correction pass, highlighting the differences with the first correction pass.

Let $\mathcal {L}'$ be the set of corrected LRS reads obtained from the first correction pass. First, graph *G*_2_ built from the *k*_2_-mers of $\mathcal {S}$ (the “Graph construction” section) is loaded in memory. Compared to *G*_1_, unitigs of *G*_2_ have a better contiguity and some of the highly branching subgraphs of *G*_1_ corresponding to repetitive regions are untangled in *G*_2_. Graph coloring and candidate SNP annotation using $\mathcal {L}'$ are performed as described in the “Graph coloring” and “Candidate SNP annotation” sections, respectively. Because the reads in $\mathcal {L}'$ are long and still erroneous in the uncorrected regions, they are not expected to be similar and Ratatosk does not perform similar reads removal.

Reads of $\mathcal {L}'$ are then anchored on the graph and non-solid regions are corrected as described in the “First correction pass” section. Parameter *B* in Eq. 1 corresponds to the size of a buffer around a non-solid region where the union of unitig colors from solid and UNSMs is computed. In the first correction pass, solid regions are expected to be short and sparse because of the high error rate of LRS reads. Hence, *B* was large enough to span two SRS reads from the same pair and the gap that intersperse them in order to capture as many colors as possible. Corrected LRS reads have no gap and are much longer than SRS reads, so it is expected that solid regions are much more abundant and contiguous than during the first correction pass. Distance *B* is therefore much smaller for the second pass (see Additional file 1) which saves computation time. Furthermore, solid regions are required to be at least *B*>*k*_2_ bases long in the second pass to increase the contiguity of solid regions and provide a better anchoring on the graph.

During path selection described in the “Traversing the graph” section, BFS traversals explored all paths of *P*_*max*_ unitigs and a path probability was assigned to each one of them before selecting one path for extension. Traversing a fixed number of unitigs avoids a combinatorial growth of the number of explored paths, especially in complex subgraphs with short cycles that are characteristic of STRs. However, as unitigs can have any length ≥*k*_1_, it has the disadvantage that the path probability might be computed for paths of *P*_*max*_ unitigs with different sequence lengths. Instead, the graph traversal in the second correction pass explores paths with a minimum sequence length of *B* bases rather than a minimum number of unitigs.

Once a path *P* has at least *B* bases in its sequence, its color matching probability *s*_*c*_ and sequence matching probability *s*_*q*_ are computed and conflated into a path probability *s*_*P*_. The construction of color set *C* used in the color matching probability *s*_*c*_ is shown in Eq. 4 and only uses the intersection of colors from each side of the non-solid region, i.e., *C*^*s*^ and *C*^*t*^, rather than the union (Eq. 1) in order to remove erroneous colors which do not belong to this region:

4 $$ \begin{aligned} C &= C^{s} \bigcup C^{t} \\ C^{s} &= \bigcap_{u \in m} C_{u} \text{,} \forall m \in M_{s}\, \textrm{ with}\,\, p_{r'}^{s} - B \leq p_{r'} \leq p_{r'}^{s} \\ C^{t} &= \bigcap_{u \in m} C_{u} \text{,} \forall m \in M_{s}\, \text{with}\,\, p_{r'}^{t} \leq p_{r'} \leq p_{r'}^{t} + B \end{aligned}   $$

### Reference-guided correction

While Ratatosk is a reference-free method, we propose an optional reference-guided preprocessing of the reads which is beneficial in several ways. This pipeline first maps the input SRS and LRS reads to a reference genome and then clusters the reads into *bins* corresponding to 5 Mbp long regions of the reference. Each bin of SRS and LRS reads is subsequently corrected independently. The benefit is three-fold:

- Graphs *G*_1_ and *G*_2_ built from an SRS bin are much smaller and contiguous than for the entire SRS data set, hence reducing the probability of selecting an incorrect path during graph traversal.
- Computation time is reduced as the search space in each bin is much smaller than for the entire SRS data set.
- Each bin is corrected independently so the workload can be distributed in parallel over many nodes of an HPC.

However, a reference-guided preprocessing also introduces some challenges. First, it is common that reference genomes contain gaps. For example, the human genome reference GRCh38.p13 has about 161 Mbp of N bases. Second, SRS reads overlapping large insertion events are expected to be unmapped. Finally, SRS reads with poor mapping qualities map ambiguously to the reference and might be incorrectly binned.

To overcome these issues, Ratatosk detects reads from the unmapped SRS reads set $\mathcal {S}_{u}$ which are likely missing in each bin. Let $\mathcal {S}_{b}$ and $\mathcal {L}_{b}$ be the subset of SRS and LRS reads of a bin *b*, respectively. To begin with, cdBGs $G^{\mathcal {S}}_{b}$ and $G^{\mathcal {L}}_{b}$ are built from the *k*_1_-mers occurring twice or more in $\mathcal {S}_{b}$ and $\mathcal {L}_{b}$, respectively. Once $G^{\mathcal {L}}_{b}$ is built, its unitigs are annotated with their mean *k*_1_-mer coverage. At first, $G^{\mathcal {L}}_{b}$ contains many more *k*_1_-mers than $G^{\mathcal {S}}_{b}$ because many erroneous *k*_1_-mers from $\mathcal {L}_{b}$ occur twice or more in the bin. To prune these erroneous *k*_1_-mers from $G^{\mathcal {L}}_{b}$, unitigs having low coverages are removed iteratively until $\left |G^{\mathcal {L}}_{b}\right | \approx \left |G^{\mathcal {S}}_{b}\right |$. Subsequently, all unmapped reads $r \in \mathcal {S}_{u}$ are queried: If *r* contains many *k*_1_-mers occurring in $G^{\mathcal {L}}_{b}$ but not in $G^{\mathcal {S}}_{b}$, *r* is suspected to be missing from the bin and is added to $\mathcal {S}_{b}$.

**Fig. 8:**
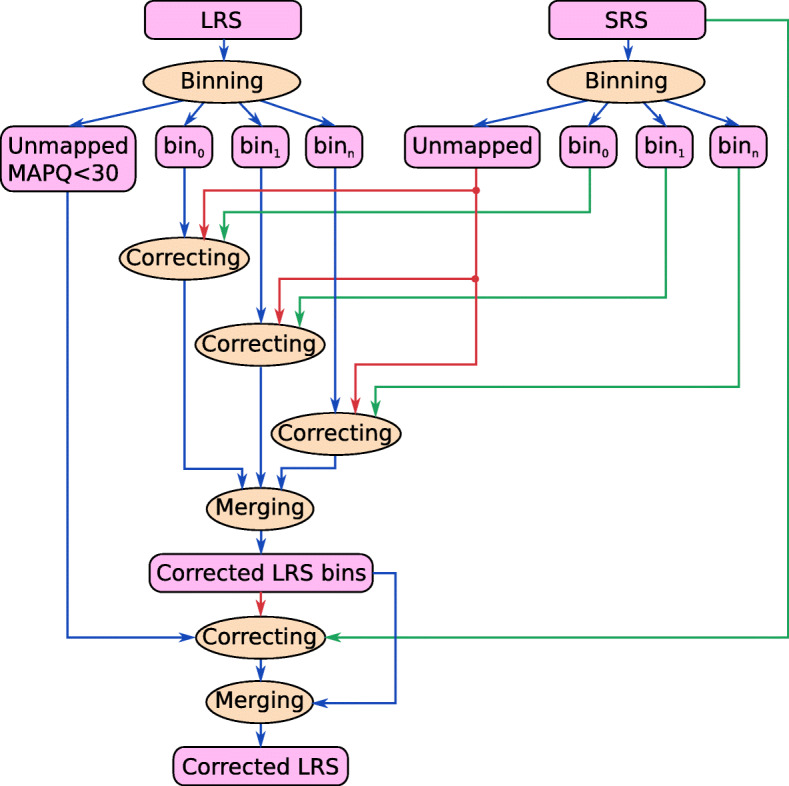
Reference-guided preprocessing of the input SRS reads (green) and LRS reads (blue). Reads are first binned and each bin is corrected independently. Unmapped or low mapping quality LRS reads are corrected using all SRS reads and all corrected LRS bins. Red arrows indicate input read sets which assist with the correction but are not corrected themselves

We outline the binning and correction pipeline proposed, as illustrated in Fig. 8, in the following. First, all reads from $\mathcal {S}$ and $\mathcal {L}$ are binned into regions of 5 Mbp according to their mapping to the reference genome. Low mapping quality (<30) and unmapped LRS reads are set aside in a bin for ambiguous long reads. Once all reads have been binned, a local correction is performed in parallel for all non-ambiguous bins and the output corrected LRS reads are merged. Note that each bin correction has access to $\mathcal {S}_{u}$ (top red arrows in Fig. 8) to retrieve the missing unmapped SRS reads from the bin. Finally, the bin of ambiguous LRS reads is corrected globally using $\mathcal {S}$. This correction is assisted by the previously corrected non-ambiguous LRS reads to enhance graph coloring during the second round of correction (bottom red arrow in Fig. 8).

## Supplementary Information

**Additional file 1** Supplementary material.

**Additional file 2** Review history.

## Data Availability

Tools: • Ratatosk v0.2 [32]: https://github.com/DecodeGenetics/Ratatoskunder the BSD-2-Clause license. • FMLRC commit 77dde49: https://github.com/holtjma/fmlrc • minimap2 v2.14-r883: https://github.com/lh3/minimap2 • Clair v2.0.6: https://github.com/HKU-BAL/Clair • rtg-tools v3.10.1: https://github.com/RealTimeGenomics/rtg-tools • Flye v2.8.1: https://github.com/fenderglass/Flye • QUAST v5.0.2: https://github.com/ablab/quast • Merqury commit ed5918c: https://github.com/marbl/merqury • purge_dups commit fe8dce2: https://github.com/dfguan/purge_dups • quast_sv_extractor.py: https://github.com/kishwarshafin/helen/tree/master/helen/modules/python/helper Ashkenazim trio data: • ONT [63]: https://precision.fda.gov/challenges/10 • PacBio [64]: https://github.com/genome-in-a-bottle/giab_data_indexes/blob/master/AshkenazimTrio/sequence.index.AJtrio_PacBio_MtSinai_NIST_subreads_fasta_10082018 • Illumina [65]: – HG002: https://ftp-trace.ncbi.nlm.nih.gov/ReferenceSamples/giab/data/AshkenazimTrio/HG002_NA24385_son/NIST_HiSeq_HG002_Homogeneity-10953946/NHGRI_Illumina300X_AJtrio_novoalign_bams – HG003: https://ftp-trace.ncbi.nlm.nih.gov/ReferenceSamples/giab/data/AshkenazimTrio/HG003_NA24149_father/NIST_HiSeq_HG003_Homogeneity-12389378/NHGRI_Illumina300X_AJtrio_novoalign_bams – HG004: https://ftp-trace.ncbi.nlm.nih.gov/ReferenceSamples/giab/data/AshkenazimTrio/HG004_NA24143_mother/NIST_HiSeq_HG004_Homogeneity-14572558/NHGRI_Illumina300X_AJtrio_novoalign_bams • Small variants v4.2 [41]: https://ftp://ftp-trace.ncbi.nlm.nih.gov/ReferenceSamples/giab/data/AshkenazimTrio/analysis/NIST_v4.2_SmallVariantDraftBenchmark_07092020/ HG002 data: • SVs* [51]: https://ftp://ftp-trace.ncbi.nlm.nih.gov/giab/ftp/release/AshkenazimTrio/HG002_NA24385_son/NIST_SV_v0.6/ • HiFi + HiCanu assembly [44]: https://ftp://ftp.dfci.harvard.edu/pub/hli/hifiasm/submission/HiCanu/HG002.HiCanu.purge.fa.gz • Ash1 v1.7 [46]: https://ftp://ftp.ccb.jhu.edu/pub/data/Homo_sapiens/Ash1/v1.7/Assembly/ Access to the raw Icelandic sequence data is available on request from Kári Stefánsson at the premises of deCODE genetics. The data are not publicly available because of Icelandic state law. * Require to be adapted from GRCh37 to GRCh38 by changing the contig names and lifting over the genomic regions to run with quast_sv_extractor.py. Access to the raw Icelandic sequence data is available on request from Kári Stefánsson at the premises of deCODE genetics. The data are not publicly available because of Icelandic state law. * Require to be adapted from GRCh37 to GRCh38 by changing the contig names and lifting over the genomic regions to run with quast_sv_extractor.py.
