# Supplementary material for: Ratatosk: hybrid error correction of long reads enables accurate variant calling and assembly
Source: Genome Biol. 2021 Jan 8;22:28. doi: 10.1186/s13059-020-02244-4 (PMC7792008; doi:10.1186/s13059-020-02244-4)
Supplement: Supplementary file 1 — Additional file 1 Supplementary material. [file 13059_2020_2244_MOESM1_ESM.pdf]

Time and memory

		FMLRC			Ratatosk		
		C	F	M	C	F	M
CPU Time (h)	T1	**6,405**	**5,044**	**5,297**	7,147	6,329	7,627
	T2	**5,671**	**5,639**	**6,419**	6,505	8,307	8,256
	T3	**6,313**	**5,327**	**4,223**	8,633	7,253	6,906
	T4	**5,189**	**4,626**	**4,921**	6,053	6,100	7,375
	HO	**4,524**	**7,872**	**8,424**	7,113	10,997	11,460
	HP	7,369	3,901	3,766	**6,723**	**3,531**	**3,206**
Memory (GB)	T1	**144.2**	**203.5**	**197.2**	260.0	317.2	295.4
	T2	**207.3**	**191.0**	**194.1**	281.1	312.4	274.2
	T3	**231.3**	**183.0**	**155.3**	319.4	278.8	270.1
	T4	**119.1**	**136.7**	**174.8**	224.8	257.0	262.8
	HO	**265.9**	**234.5**	**246.0**	292.2	286.1	287.8
	HP	**265.9**	**234.5**	**266.8**	292.5	282.4	283.0

Input data preprocessing accounts for a negligible amount of time and memory compared to the total running time. Ratatosk preprocessing of the HG002 ONT data set took 143 CPU hours and 29.8 GB of memory while FMLRC preprocessing took 25 CPU hours and 74.5 GB of memory.

Ratatosk was run in parallel on several machines due to the reference-guided preprocessing of the input data while FMLRC was run on a single machine. FMLRC and the ambiguous bin correction of Ratatosk were run with 48 threads on machines with 350 GB of RAM available. Ratatosk non-ambiguous bin corrections were run with 8 threads on machines with 16 GB of RAM available. The reported peak of memory for Ratatosk matches the correction of the ambiguous LRS bin at the end of the preprocessing pipeline as it requires to use all the input SRS data. Indeed, Ratatosk memory usage is dominated by the graph index and the SRS data coloring of its vertices during the first correction pass.

Error rate

Let r be an LRS read which has been aligned to a reference genome. We define the following:

- $|r|$: Number of bases in r
- D_r: Number of deleted bases in alignment of r to reference
- I_r: Number of inserted bases in alignment of r to reference
- M_r: Number of mismatching bases in alignment of r to reference
- S_r: Number of soft-clipped bases in alignment of r to reference

The deletion, insertion and substitution error rates of a set of LRS reads $\mathcal{L}$ are:

$$\begin{aligned} E_D &= \sum_{r \in \mathcal{L}} \frac{D_r}{|r| - S_r} \\ E_I &= \sum_{r \in \mathcal{L}} \frac{I_r}{|r| - S_r} \\ E_M &= \sum_{r \in \mathcal{L}} \frac{M_r}{|r| - S_r} \end{aligned} \tag{1}$$

And the combined error rate of R is:

$$E = \sum_{r \in \mathcal{L}} \frac{D_r + I_r + M_r}{|r| - S_r} \tag{2}$$

The error rates are computed from primary alignments only.

Ambiguous bases

Ambiguous bases are bases which soft-clip in the primary alignment but map in non-overlapping supplementary alignments to distant reference positions, such as different chromosomes, compared to the primary alignment reference position. The ratio of ambiguous bases aim to measure the proportion of over-corrected bases in reads. To avoid counting ambiguous bases in supplementary alignments of a read r that might correspond to a large SV event, a supplementary alignment is only used if it does not overlap the primary alignment nor another supplementary alignment of r with a large buffer of 1 Mbp on each side of the alignment. Algorithm 1 details the ambiguous bases ratio computation for a read represented by a primary alignment and a set of supplementary alignments.

Algorithm 1 Compute ratio of ambiguous bases

Input: Read r, Primary alignment P, list of supplementary alignments $\mathcal{S}$

function AmbiguousBases(r, P, $\mathcal{S}$)
- $\mathcal{S}' \leftarrow \mathsf{sort}(\mathcal{S})$ ▷ Sort by decreasing alignment score
- $\mathcal{C}_a \leftarrow \varnothing$ ▷ Set of ambiguous read positions
- $\mathcal{C}_P \leftarrow$ set of soft-clipped read positions in P
- $B \leftarrow 1{,}000{,}000$ ▷ Buffer size in bp
- $T \leftarrow$ empty interval tree
- $p_s^P \leftarrow$ first mapped position of P in reference
- $p_e^P \leftarrow$ last mapped position of P in reference
- $T.\mathsf{addInterval}(p_s^P, p_e^P)$ ▷ Add $[p_s^P, p_e^P]$
- **for each** $S \in \mathcal{S}'$ **do**
  - $p_s^S \leftarrow$ first mapped position of S in reference
  - $p_e^S \leftarrow$ last mapped position of S in reference
  - $\mathcal{O} \leftarrow T.\mathsf{getOverlappingIntervals}(p_s^S - B, p_e^S + B)$
  - **if** $|\mathcal{O}| = 0$ **then** ▷ No overlap
    - $\mathcal{M}_S \leftarrow$ set of mapped read positions in S
    - $\mathcal{C}_a \leftarrow \mathcal{C}_a \cup (\mathcal{C}_P \cap \mathcal{M}_S)$
    - $T.\mathsf{addInterval}(p_s^S, p_e^S)$
- **return** $\frac{|\mathcal{C}_a|}{|r|}$

Subsampling

	Raw			Ratatosk		
	HG002	HG003	HG004	HG002	HG003	HG004
Mean error rate (%)	8.81	7.82	8.24	**1.41**	**1.39**	**1.30**
Aligned reads (%)	42.70	60.34	46.48	**43.03**	**60.75**	**46.81**
Ambiguous bases (%)	0.46	0.50	0.40	**0.41**	**0.44**	**0.36**
SNP F1 (%)	70.78	78.41	72.69	**87.91**	**87.79**	**88.13**
Indel F1 (%)	25.24	32.36	26.38	**75.52**	**74.39**	**77.19**

(a) 10x ONT and 30x Illumina

	Raw			Ratatosk		
	HG002	HG003	HG004	HG002	HG003	HG004
Mean error rate (%)	8.81	7.83	8.24	**1.41**	**1.39**	**1.30**
Aligned reads (%)	42.70	60.33	46.47	**43.03**	**60.74**	**46.81**
Ambiguous bases (%)	0.46	0.50	0.41	**0.41**	**0.44**	**0.37**
SNP F1 (%)	91.86	94.76	92.85	**95.91**	**96.37**	**96.19**
Indel F1 (%)	40.16	45.96	41.41	**84.39**	**82.96**	**84.73**

(b) 20x ONT and 30x Illumina

	Raw			Ratatosk		
	HG002	HG003	HG004	HG002	HG003	HG004
Mean error rate (%)	8.81	7.82	8.25	**1.41**	**1.39**	**1.30**
Aligned reads (%)	42.70	60.31	46.47	**43.03**	**60.73**	**46.81**
Ambiguous bases (%)	0.46	0.50	0.41	**0.41**	**0.44**	**0.37**
SNP F1 (%)	96.36	97.64	96.89	**97.22**	**97.53**	**97.47**
Indel F1 (%)	46.88	51.79	48.19	**87.22**	**85.72**	**87.44**

(c) 30x ONT and 30x Illumina

Small variants were called with Clair using the models trained on the HG002 46.72x ONT and HG003 85.23x ONT data sets. The optimal quality scores computed by rtg-tools were used for filtering.

RTG-tools quality score thresholds

		Threshold
HG002	Raw	770
	FMLRC	683
	Ratatosk	523
HG003	Raw	789
	FMLRC	751
	Ratatosk	511
HG004	Raw	754
	FMLRC	677
	Ratatosk	509

Command lines

- Ratatosk
  - Reference-guided

```
$ bash Ratatosk.sh [REFERENCE].fa [INPUT_SHORT_READS].bam
[INPUT_LONG_READS].bam [OUTPUT]
```

  - *de novo*

```
$ Ratatosk -c [THREADS] -s [INPUT_SHORT_READS].fq -l
[INPUT_LONG_READS].fq -o [OUTPUT]
```

- FMLRC

```
$ awk 'NR%4==2' [INPUT_SHORT_READS].fq | sort -S50G
--parallel=[THREADS] > [INPUT_SHORT_READS].sort.fq
$ cat [INPUT_SHORT_READS].sort.fq | tr NT TN | ropebwt2 -LR |
tr NT TN | fmlrc-convert [INPUT_SHORT_READS].npy
$ fmlrc -p [THREADS] [INPUT_SHORT_READS].npy
[INPUT_LONG_READS].fq [OUTPUT]
```

- Flye

```
$ flye --threads [THREADS] --nano-corr [INPUT_LONG_READS].fq
--out-dir [OUTPUT]
```

Parameter `--nano-raw` was used instead of `--nano-corr` for the assembly of the raw ONT reads.

- minimap2

```
$ minimap2 -t [THREADS] -ax map-ont -Y [REFERENCE].fa
[INPUT_LONG_READS].fq > [INPUT_LONG_READS].sam
```

Parameter `map-pb` was used instead of `map-ont` for the mapping of the PacBio reads.

- Clair

```
$ python clair.py callVarBam --chkpnt_fn [MODEL] --ref_fn
[REFERENCE].fa --bam_fn [INPUT_LONG_READS].bam --ctgName
[CHROMOSOME] --sampleName [ID] --call_fn [OUTPUT].vcf
--threads [THREADS]
```

- rtg-tools

```
$ rtg vcfeval -e [GIAB_TRUTHSET_SMALLVAR].bed -b
[GIAB_TRUTHSET_SMALLVAR].vcf -c [CLAIR_SMALLVAR_CALLS].vcf
-o [OUTPUT] -t [REFERENCE].sdf
```

- Quast

```
$ python quast.py -t [THREADS] -s --large -r [REFERENCE].fa
-o [OUTPUT] [ASSEMBLY].fa
$ python quast_sv_extractor.py -c GRCh38_centromere_gaps.bed
-d GRCh38_masked_regions.bed -s Tier1plusTier2_hg38_v0.6.1.bed
-q contigs_reports/all_alignments*broken.tsv
```

- Merqury

```
$ bash merqury.sh [INPUT_SHORT_READS].meryl [ASSEMBLY].fa
[OUTPUT]
```

- purge_dups

```
$ minimap2 -t [THREADS] -xmap-ont -I6G [ASSEMBLY].fa
[INPUT_LONG_READS].fq > [OUTPUT].paf
$ pbcstat [OUTPUT].paf
$ calcuts PB.stat > cutoffs 2> calcuts.log
$ split_fa [ASSEMBLY].fa > [OUTPUT].split
$ minimap2 -t [THREADS] -xasm5 -I6G -DP [OUTPUT].split
[OUTPUT].split > [OUTPUT].split.paf
$ purge_dups -2 -T cutoffs -c PB.base.cov [OUTPUT].split.paf
> dups.bed 2> purge.log
$ get_seqs dups.bed [ASSEMBLY].fa
```

Default parameters

Graph construction and coloring:

- $k_1 = 31$
- $k_2 = 63$
- $F = 0.25$
- $T_{max}(u) = 512 \cdot (|u| - k + 1)$ for $u \in V$

First correction pass:

- $T_{min} = 2$
- $B = 500$
- $D = 0.1$
- $P_{max} = 4$

Second correction pass:

- $T_{min} = 1$
- $B = 95$

Graph coloring

Ratatosk uses hashing to ensure that two reads from the same pair get the same color. Each read is initially given an identifier which is the hash of the read name and each identifier is associated with a color. To simplify the coloring and curb its running time, pair identifiers are not guaranteed to be unique such that multiple read pairs might hold the same identifier, hence the same color, because their read names hash to the same value. These collisions are random and are expected to have little impact on the correction. Ratatosk enables a memory efficient graph coloring by using two techniques. First, it discards *similar* read pairs from the coloring as they represent redundant information to index, especially in the case of high coverage SRS data. Second, Ratatosk compacts the set of colors assigned to each unitig by using consecutive color values rather than random values. Consecutive color values can be compacted in memory using Run Length Encoding, delta encoding and compacted bitmaps (1). The graph coloring objective is for each read pair $P_c \in \mathcal{S}$ to color a set of unitigs $\mathcal{U}_c \in G_1$ with color c. Each unitig $u \in \mathcal{U}_c$ shares at least one k_1-mer with P_c. We devised a probabilistic algorithm which colors the graph while discarding similar read pairs and assigning consecutive color values to unitigs in a greedy fashion. The algorithm performs two steps: A partial coloring of the graph is initially made for read pair filtering and color compaction purposes before completing a full graph coloring. In the partial coloring, each read pair P_c will only color the longest unitig $u \in \mathcal{U}_c$. This unitig acts as a centroid in the graph and it is expected that read pairs similar to P_c will color the same unitig. Hence, color c and a hash $h(\mathcal{U}_c)$ are assigned to unitig u unless there exist another pair similar to P_c which has already been assigned to u. Two read pairs P_c and P'_c are similar if they have the same unitig set hash, i.e , $c \neq c'$ but $h(\mathcal{U}_c) = h(\mathcal{U}_{c'})$. In which case, P_c is discarded and will not be used for the full graph coloring. Next, read pairs are given new colors by assigning, when possible, consecutive color values to pairs clustering to the same unitigs. A final graph coloring is then performed using read pairs which are not discarded and their new color values. The mean k_1-mer coverage of unitigs is also computed during the final graph coloring. After coloring the graph, all unitigs with a mean k_1-mer coverage lower than a pre-defined threshold T_{min} (see Additional file 1) are removed from the graph. In order to limit even further the memory usage of Ratatosk, unitigs having a mean k_1-mer coverage greater than T_{max} have their colors discarded. These unitigs are usually k_1-mers with a low sequence entropy such as poly-$\{A, C, G, T\}$ or k_1-mers occurring within STRs. They are typically located within highly branching subgraphs and their colors provide little guidance information while severely impacting the running time.

References

[1] Samy Chambi, Daniel Lemire, Owen Kaser, and Robert Godin. Better bitmap performance with Roaring bitmaps. *Software: Practice and Experience*, 46(5):709–719, 2016.
