# Supplementary material for: Ratatosk: hybrid error correction of long reads enables accurate variant calling and assembly
Source: Genome Biol. 2021 Jan 8;22:28. doi: 10.1186/s13059-020-02244-4 (PMC7792008; doi:10.1186/s13059-020-02244-4)
Supplement: Supplementary file 2 — Additional file 2 Review history. [file 13059_2020_2244_MOESM2_ESM.docx]

Review History

**First round of review**

**Reviewer 1**

**Are you able to assess all statistics in the manuscript, including the appropriateness of statistical tests used?**

Yes

**Comments to author:**

Holley et al present a paper which describes a method to error correct genomic ONT reads with the help of short read data. Such methods are called hybrid error correctors and, unless/until ONT error rates have a substantial drop, are a powerful way to improve the usability of ONT data. The need for such methods are clear: ONT data is increasingly popular and will be used in a vast number of future studies. Error correction will remain necessary, even if the error rates continue to drop. Thus this paper addresses an important need of the life science community. Their method uses several novel algorithmic techniques and is able to successfully error correct a whole human >50x coverage ONT dataset, albeit using a lot of computational resources. Nevertheless, the accuracy results are potentially impressive (subject to some of my comments below) and the tool is likely to be widely used by the community.

Major comments

* The title and many other places refer to Ratatosk as a tool for long read correction. However, it is only evaluated on ONT data. In principle, it could be possible that Ratatosk might also work for PacBio data. But, without any evaluation, it might be misleading to call it a long read corrector rather than a ONT read corrector. I suggest for the authors to modify the manuscript (especially the title and abstract) to always be specific that the long read data which Ratatosk corrects is ONT data. I suggest replacing "long reads" throughout the manuscript with "ONT reads."

* In a similar vein, the authors should make clear that their tool is for genomic, rather than transcriptomic reads. The two types of data are quite different and it is unlikely that a genomic corrector would work for transcriptomic reads (as was shown in Lima et al. https://doi.org/10.1093/bib/bbz058). Currently, the title and abstract make one think that it could work for both kinds of data.

* The time and memory results are missing important details. What were the parameters of the machine? How many CPUs/threads were used?

* The time and memory results are not very good, though given the improvements in accuracy over other tools, and the fact that other tools are not very fast either, I think they are acceptable. However, I do think it is an important point for anyone trying to use the tool. I suggest that the authors say a little more in the main text to acknowledge that this is a current shortcoming of the tool and that they are in the process of improving it (as they indicate in the Supplementary already).

* How does the tool perform, in comparison to others, with lower coverage of ONT data? I suggest that the authors design down-sampling experiments on their datasets to test this. The ONT coverages currently tested on (>50x) might be unrealistic in some settings (e.g. for a resequencing experiment to call small variants).

* For Table 2, what is the 1) number of reads output, and 2) the percentage of reads aligned? These are important metrics to consider because a tool could simply discard erroneous reads to get better error rates or it could actually miscorrect a lot of reads. Adding these two metrics I think is important to truly judge the relative performances of the tools.

* The authors are well justified to compare against FMLRC, which generally outperforms other tools in the surveys of Fu et al and Zhang et al. However, those surveys only benchmark organisms much simpler than humans (e.g. bacteria and yeast). The survey by Morisse et al., on the other hand, does evaluate on human ONT data for a single chromosome. In that comparison, FMLRC is outperformed by hg-color. This seems to indicate that for human size data, hg-color might be the tool to beat. Could the authors add hg-color in their evaluation? I also think that having a second tool in the comparison, besides FMLRC, will make the results of the paper much more robust.

*It says that a model was trained with Clair on HG002-1 dataset, but then the same model was used by Clair to call reads on the same dataset for the purposes of evaluation. Unless I am misunderstanding what was done, this would be equivalent to using the same training set as the validation set, which would allow Clair to overfit the data. This makes the result of Table 3 hard to interpret and leaves open the question of the SNP/Indel calling accuracy that would be achieved by Ratatosk and FMLRC in practice. I suggest that the authors address this in the text by using a training dataset that is different from the evaluation one. If there are no other datasets that can be used to achieve this, why not train the model on one chromosome and to the validation on the other chromosomes? (because the read set is human, reads could be pre-filtered by mapping according to chromosomes, so this should be feasible.)

* For the assembly (Table 5), could the authors compare to running Flye on the raw reads? Currently the table is demonstrating the feasibility of assembly with ONT data, which is already known. What would be more relevant is to see that running Ratotsk as pre-processing actually improves assembly quality vs. not running it.

Minor comments

* The first sentence of the paper refers to "Ratatoskr." My knowledge of Norse mythology leaves everything to be desired, but is this a typo -- should it be Ratotsk without the r in the end?

* The sentence on page 3 line 24 (Overall, hybrid correction methods...." needs a citation.

* I could not find the download information for the 4 Icelandic trios. I assume that this data is publicly available (otherwise I would ask for evaluations to be done on publicly available data). Please provide an accession number or something similar.

* "Path P is said non-branching" --> "Path P is said to be non-branching"

* I was a bit lost in the section on Graph Coloring. What is the goal here? Is it to assign a color to each read pair and a set of colors to each unitig so that this set corresponds to all the read pairs that contain a k-mer in that unitig? If that's the case, then its very simple, and the procedure the authors describe in the first paragraph does the trick. But the section continues...as far as I understand, the authors are trying to further optimize this coloring. The optimization, from what I can tell, is to reduce the total number of colors while maintaining some property which is not very clear to me. Can the objective be spelled out more explicitly in the beginning of the section?

* The authors do a good job of citing related work, but the one part that remained unclear to me was how the method here relates to the previous work. The one I am most familiar with is Lordec. From what I gathered, Ratatosk is a more sophisticated version of Lordec, with at leat one key addition. During path extension / gap filling, Ratatosk not only tries to find a path using solid kmers, but tries to make sure the reads completing that path are similar to the ones in the solid regions and consistent with each other. I am not sure if this assessment is correct. Basically, the question I have is: Methodologically, what is the key idea or ideas that make this work and that the reader can take away and possibly apply to other projects? I think that if the authors can say something with respect to this, it would make the paper more impactful to algorithm developers.

**Reviewer 2**

**Are you able to assess all statistics in the manuscript, including the appropriateness of statistical tests used?**

Yes

**Comments to author:**

Holley et al. present a method for hybrid long-read error correction that is implemented in a tool "Ratatosk". In contrast to prior work, this method is designed to specifically avoid over-correction by leveraging both the low-error rates of short read sequencing (SRS) in an initial pass, and then the contiguity of long read sequencing (LRS) in a second pass. The method uses colored compact de Bruijn Graphs (ccdBG) to represent the data in k-mer space (with two different values for k). Additionally, Holley et al. list four specific features which I will refer to in my comments:

1. SRS and LRS are both used to color the ccdBGs throughout the process

2. Near-match k-mers (specifically, off-by-one) are used while anchoring LRS

3. SNPs are annotated to the ccdBG

4. Two k-mers passes are used, leveraging the advantages of SRS and LRS differently in the two passes

Also a fifth feature of significance, although not highlighted in the paper:

5. Can use a reference-guided approach; this is of particular importance as all results presented appear to be from the reference-guided method

Holley et al. focus their results on a correction comparison and show downstream results using an assembly comparison (using Flye) in the context of the human genome. For the correction comparison, they compare to FMLRC. In general, the results of Ratatosk seems to outperform FMLRC (with a larger run-time cost) on error correction. It also seems to create corrected reads that are better suited for variant calling, which is not too surprising given that FMLRC does not appear to have been specifically designed with that use case in mind. For assembly, they compare to results from a HiFi and HiCanu assembly as well as a recent Ash1 assembly. The results seem to be generally comparable to those assemblies with the best results in a few of the metrics.

Overall, the results are quite promising, especially in the context of diploid (e.g. human) genomes. The algorithmic/data structure usage is also of broad interest, and it seems like Ratatosk may further enable downstream analyses (compared to other hybrid correctors) that are currently still challenging. Additionally, the tool was downloaded and compiled following the instructions provided via GitHub (note: I did not run the method on any data).

However, the manuscript could benefit from some more details regarding the impact of the five features above, especially the reference-guided approach. Additionally, while reading the manuscript, I had a few questions around the process used to generate results that do not seem to be answered in the main document or an appendix/supplement.

Major Comments:

1. Of the five features, the optional use of the reference-guided approach is potentially the most consequential, and simultaneously the least mentioned in the manuscript. This is of particular importance in the context of de novo assembly where a reference (even a low-quality reference) is frequently unavailable. While the method is designed to avoid overcorrection, the addition of a reference creates the possibility of reference bias in the results (note, I believe this is not very likely given the results from vcfeval). The authors also state that the reference guidance impacts both the correction and the computation time in the methods. Given that this approach is optional in Ratatosk, it seems appropriate that the results from both the reference-guided approach and the reference-free approach should be included in this document. Assuming there is an impact on the results (including downstream assemblies) and computation time, it should be discussed as well. To some readers, this may also present a more realistic or "fair" comparison to tools that did not assume the presence of a reference, both for correction and downstream assembly.

2. Of the other four features, feature 4 has been discussed in previous papers (FMLRC at least, possible others; Wang et al., Table 1), but to my knowledge 1-3 have not. Feature 2 seems to be generally applicable to k-mer based correction, whereas features 1 and 3 seem to be more specific to ccdBG. It would be beneficial to discuss the impact that 1-3 each had on the corrected reads to give the reader a greater understanding of how much benefit comes from using the ccdBG data structure (i.e features 1 & 3) v. using a better method during read anchoring/correction (i.e. feature 2). Providing data/evidence to support this discussion would be ideal (as previous works have done with feature 4), but these features do not appear to be easily disabled by command-line parameters in Ratatosk.

3. The datasets used for testing use a mix a guppy v3.3 and v3.6, but most were generated with v3.3. The authors state that v3.6 offers better base-level accuracy. Presumably, the raw data for the Icelandic trios is available to the authors. If this is the case, why is mostly v3.3 data used? It adds a layer of complexity to the manuscript that seems to be avoidable.

4. The authors suggest that "FMLRC is prone to overcorrection" after presenting the comparison results, but they don't seem to perform an equivalent comparison in terms of parameter selection (where available, of course). For example, Ratatosk appears to use a lower minimum threshold on "solid" (Tmin=2 or 1, depending on the pass) than FMLRC (assuming default, m=5). Given the sample coverage and the use case, the setting from Ratatosk is likely more appropriate (FMLRC appears to have been tested on non-human data with much higher coverage), but it is unclear how much of an impact that would have on the perceived overcorrection. Theoretically, the higher threshold on FMLRC would reduce the number of "solid" paths available for correction, so some of the over-corrections could just be a result of poor parameter choice for this use case. Additionally, the k-mer sizes in the two passes appear to be different with Ratatosk using longer k-sizes overall. The longer k-sizes are likely of benefit to Ratatosk given the inexact matching scheme, but may hinder FMLRC since it only uses exact solid matches. A more equivalent comparison would be ideal (i.e. identical or closer thresholds and k-sizes), but running that experiment is likely computationally expensive.

Instead, I suggest the authors specifically point out that these similar parameters are set differently and discuss how that may impact the comparison. If there are other comparable parameters aside from solid threshold and k-sizes, they should be included as well. Additionally, it would be beneficial to explain their choice of parameters, especially given that many other correction algorithms (including those not tested here) use a smaller k-size by default (17, 19, and 21 seem to be relatively common). Any evidence to support their parameter choices would also be beneficial in a supplement/appendix, even if only tuned using a subset of their data.

5. For the correction analysis, what were the file types used for upstream alignment? Ratatosk seems to be capable of producing FASTQ files with quality scores, which has been noted to greatly benefit alignment and variant calling steps downstream. It's unclear what was used for Raw, while FMLRC only seems to produce FASTA files. Which file types were used should be stated clearly for the reader. If the FASTQ files were used in Ratatosk, it should be highlighted as a (likely) benefit of using Ratatosk over FMLRC and other tools that do not produce quality scores, and also noted as something likely potentially hindering the aligner/caller on FMLRC results.

6. For the assembly comparison, how was Quast run? I assume a reference was used, and presumably it was hg38 based on the reference used during alignment. These should be stated.

7. (I'm assuming the answer to the above comment is that it was compared to hg38) When reviewing the assembly results, I found it odd that the indels per 100kbp are all roughly the same while the mismatches per 100kbp are much lower from the Ratatosk/Flye assembly than either of the other two assemblies, especially given that Ash1 includes hg38 patches and that Ratatosk is designed to avoid overcorrection (i.e. preserve variation). Is there some justification for this discrepancy, or do the authors believe the entire difference is from error correction? If from correction, why is it not impacting indels the same way? Is this possibly a reference bias injected from reference-guidance? (This last question could be answered in part with reference-free results)

Minor Comments:

1. I was unable to locate any settings used for FMLRC, Clair, Flye, Quast, or Merqury (versions are in the Appendix). These should be stated or "default" if no parameters were changed. Preferably, the exact commands for all steps of processing/analysis would be available in an appendix/supplement. Additionally, default parameters for Ratatosk are stated in the appendix. Were these the ones used to generate all results?

2. For table 3a, it would be beneficial to know what the selected "optimal" quality score is for each method (assuming they are different).

3. The GIAB version should be stated in the main text (it appears to be 3.3.2 from the Appendix).

4. The pre-processing requirements (CPU/memory) for Ratatosk should be listed separately under the Time and Memory section (even if comparably negligible) for completion. If available, it would likely be beneficial for FMLRC requirements as well for comparison (again, even if comparably negligible).

5. If the Icelandic trio data is publicly available (it may not be), it should be listed in Appendix F.

Suggestions:

1. The authors may want to consider greater emphasis on the fact that their tests show Ratatosk working well on human datasets. Both reviews mentioned (Fu et al. and Zhang et al.) focused their benchmarks on relatively small non-human organisms (E. coli, yeast, etc), and neither seems to directly assess the impact on variant calling. To my knowledge, the tools evaluated in those benchmarks do not directly address these problems either (most were focused on de novo assembly, and preserving variation was likely secondary if considered at all). This would allow the authors to emphasize how the Ratatosk features address complexity of larger genomes, especially where preserving variation matters.

**Authors Response**

**Point-by-point responses to the reviewers’ comments:**

Reviewer 1

Holley et al present a paper which describes a method to error correct genomic ONT reads with the help of short read data. Such methods are called hybrid error correctors and, unless/until ONT error rates have a substantial drop, are a powerful way to improve the usability of ONT data. The need for such methods are clear: ONT data is increasingly popular and will be used in a vast number of future studies. Error correction will remain necessary, even if the error rates continue to drop. Thus this paper addresses an important need of the life science community. Their method uses several novel algorithmic techniques and is able to successfully error correct a whole human >50x coverage ONT dataset, albeit using a lot of computational resources. Nevertheless, the accuracy results are potentially impressive (subject to some of my comments below) and the tool is likely to be widely used by the community.

Major Comments:

* The title and many other places refer to Ratatosk as a tool for long read correction. However, it is only evaluated on ONT data. In principle, it could be possible that Ratatosk might also work for PacBio data. But, without any evaluation, it might be misleading to call it a long read corrector rather than a ONT read corrector. I suggest for the authors to modify the manuscript (especially the title and abstract) to always be specific that the long read data which Ratatosk corrects is ONT data. I suggest replacing "long reads" throughout the manuscript with "ONT reads."

*Author' answer: Ratatosk was only evaluated on Oxford Nanopore data as in practice, this sequencing platform is the one that benefits the most from the hybrid correction using Illumina reads. Illumina correction of PacBio reads is less relevant because PacBio already offers very accurate long reads ("HiFi") using a circular sequencing technology. Yet, we agree with the reviewer's comment and the evaluation of Ratatosk was updated with an additional data set of PacBio CLR reads for HG002, HG003 and HG004.*

* In a similar vein, the authors should make clear that their tool is for genomic, rather than transcriptomic reads. The two types of data are quite different and it is unlikely that a genomic corrector would work for transcriptomic reads (as was shown in Lima et al. https://doi.org/10.1093/bib/bbz058). Currently, the title and abstract make one think that it could work for both kinds of data.

*Author' answer: The introduction was updated to make it clear that Ratatosk applies only to genomic long reads. Furthermore, a reference to Lima et al., 2019 was added to Section 1.1.*

* The time and memory results are missing important details. What were the parameters of the machine? How many CPUs/threads were used?

*Author' answer: These details are now reported in the Appendix of the revised manuscript.*

* The time and memory results are not very good, though given the improvements in accuracy over other tools, and the fact that other tools are not very fast either, I think they are acceptable. However, I do think it is an important point for anyone trying to use the tool. I suggest that the authors say a little more in the main text to acknowledge that this is a current shortcoming of the tool and that they are in the process of improving it (as they indicate in the Supplementary already).

*Author' answer: The Appendix of the revised manuscript lists the CPU time and memory requirement for each tool and data set. All data sets were re-corrected with Ratatosk v0.2, compared to the v0.1 in the submitted manuscript, which improves the running time. The Result section of the revised manuscript mentions that FMLRC is 28 % faster and consumes 39 % less memory than Ratatosk. The Conclusion of the revised manuscript acknowledges that this is a shortcoming of the current version but improvements are possible.*

* How does the tool perform, in comparison to others, with lower coverage of ONT data? I suggest that the authors design down-sampling experiments on their datasets to test this. The ONT coverages currently tested on (>50x) might be unrealistic in some settings (e.g. for a resequencing experiment to call small variants).

*Author' answer: The raw ONT reads of the Ashkenazim trio were subsampled to 10x, 20x and 30x coverage. These reads were then corrected with Ratatosk using Illumina reads downsampled at 30x coverage. Results are reported in the Appendix and discussed in the Results section.*

* For Table 2, what is the 1) number of reads output, and 2) the percentage of reads aligned? These are important metrics to consider because a tool could simply discard erroneous reads to get better error rates or it could actually miscorrect a lot of reads. Adding these two metrics I think is important to truly judge the relative performances of the tools.

*Author' answer: As neither Ratatosk nor FMLRC discards reads from their output, we enclosed in the Results section of the revised manuscript a table showing for each data set the ratio of aligned reads compared to the total number of raw reads.*

* The authors are well justified to compare against FMLRC, which generally outperforms other tools in the surveys of Fu et al and Zhang et al. However, those surveys only benchmark organisms much simpler than humans (e.g. bacteria and yeast). The survey by Morisse et al., on the other hand, does evaluate on human ONT data for a single chromosome. In that comparison, FMLRC is outperformed by hg-color. This seems to indicate that for human size data, hg-color might be the tool to beat. Could the authors add hg-color in their evaluation? I also think that having a second tool in the comparison, besides FMLRC, will make the results of the paper much more robust.

*Author' answer: HG-Color is more adapted to correct smaller genomes than human genomes. Indeed, its long running time makes it unpractical to run for even a single genome of our experiments. The review of Morisse et al. shows that HG-Color takes about a week (wall-clock time) to correct 30x ONT reads from a single chromosome. The main author of HG-Color estimates that it would take up to one year to correct a full genome (https://github.com/morispi/HG-CoLoR/issues/18). Furthermore, while HG-Color shows the best performance in the Remapping category of the Morisse et al. review, it is outperformed by FMLRC in the Assembly category. Hence, HG-Color was not included in our experiments because of the two aforementioned reasons.*

* It says that a model was trained with Clair on HG002-1 dataset, but then the same model was used by Clair to call reads on the same dataset for the purposes of evaluation. Unless I am misunderstanding what was done, this would be equivalent to using the same training set as the validation set, which would allow Clair to overfit the data. This makes the result of Table 3 hard to interpret and leaves open the question of the SNP/Indel calling accuracy that would be achieved by Ratatosk and FMLRC in practice. I suggest that the authors address this in the text by using a training dataset that is different from the evaluation one. If there are no other datasets that can be used to achieve this, why not train the model on one chromosome and to the validation on the other chromosomes? (because the read set is human, reads could be pre-filtered by mapping according to chromosomes, so this should be feasible.)

*Author' answer: In the revised manuscript, a model was trained independently for HG002, HG003 and HG004 for each raw and corrected data set. Subsequently, small variants of HG002 and HG004 were called with the model trained on HG003 while the small variants of HG004 were called with the model trained on HG002.*

* For the assembly (Table 5), could the authors compare to running Flye on the raw reads? Currently the table is demonstrating the feasibility of assembly with ONT data, which is already known. What would be more relevant is to see that running Ratotsk as pre-processing actually improves assembly quality vs. not running it.

*Author' answer: The revised manuscript now includes the raw data assembly with Flye. Note that the Flye and Ratatosk versions used in the revised manuscript differ from the submitted manuscript (previously 2.7 and 0.1 respectively, now updated to 2.8 and 0.2).*

Minor Comments:

* The first sentence of the paper refers to "Ratatoskr." My knowledge of Norse mythology leaves everything to be desired, but is this a typo -- should it be Ratotsk without the r in the end?

*Author' answer: Ratatoskr is the original name of the mythological squirrel as it appears in the Snorra-Edda. To ease the pronunciation of the tool name for non-Nordic languages, the last character was removed from the tool name.*

* The sentence on page 3 line 24 (Overall, hybrid correction methods...." needs a citation.

*Author' answer: A citation has been added to the revised manuscript.*

* I could not find the download information for the 4 Icelandic trios. I assume that this data is publicly available (otherwise I would ask for evaluations to be done on publicly available data). Please provide an accession number or something similar.

*Author' answer: Access to the raw Icelandic sequence data is available on request from Kári Stefánsson at the premises of deCODE genetics. The data are not publicly available because of Icelandic state law. This is now mentioned in the Data section of the revised manuscript. However, all of our experiments are performed and are fully reproducible using the Ashkenazim trio from Genome In A Bottle.*

* "Path P is said non-branching" to "Path P is said to be non-branching"

*Author' answer: Fixed.*

* I was a bit lost in the section on Graph Coloring. What is the goal here? Is it to assign a color to each read pair and a set of colors to each unitig so that this set corresponds to all the read pairs that contain a k-mer in that unitig? If that's the case, then its very simple, and the procedure the authors describe in the first paragraph does the trick. But the section continues...as far as I understand, the authors are trying to further optimize this coloring. The optimization, from what I can tell, is to reduce the total number of colors while maintaining some property which is not very clear to me. Can the objective be spelled out more explicitly in the beginning of the section?

*Author' answer: The reviewer correctly summarized the goal of the graph coloring. We added one extra sentence at the beginning of the second paragraph to explain why memory optimizations are required. The memory optimization descriptions have been moved to the Appendix as they are not essential to understand the remaining methods and algorithms.*

* The authors do a good job of citing related work, but the one part that remained unclear to me was how the method here relates to the previous work. The one I am most familiar with is Lordec. From what I gathered, Ratatosk is a more sophisticated version of Lordec, with at leat one key addition. During path extension / gap filling, Ratatosk not only tries to find a path using solid kmers, but tries to make sure the reads completing that path are similar to the ones in the solid regions and consistent with each other. I am not sure if this assessment is correct. Basically, the question I have is: Methodologically, what is the key idea or ideas that make this work and that the reader can take away and possibly apply to other projects? I think that if the authors can say something with respect to this, it would make the paper more impactful to algorithm developers.

*Author' answer: The Introduction contains a paragraph describing what are the 5 key features introduced in Ratatosk and not included in other hybrid correction tools. For more clarity, these key points are re-stated in the Conclusion of the revised manuscript. We also change the description of the existing tools to mention that most hybrid correction methods for long reads, including Ratatosk, extend the core ideas of Lordec.*

Reviewer 2

Holley et al. present a method for hybrid long-read error correction that is implemented in a tool "Ratatosk". In contrast to prior work, this method is designed to specifically avoid over-correction by leveraging both the low-error rates of short read sequencing (SRS) in an initial pass, and then the contiguity of long read sequencing (LRS) in a second pass. The method uses colored compact de Bruijn Graphs (ccdBG) to represent the data in k-mer space (with two different values for k). Additionally, Holley et al. list four specific features which I will refer to in my comments:

- SRS and LRS are both used to color the ccdBGs throughout the process

- Near-match k-mers (specifically, off-by-one) are used while anchoring LRS

- SNPs are annotated to the ccdBG

- Two k-mers passes are used, leveraging the advantages of SRS and LRS differently in the two passes

Also a fifth feature of significance, although not highlighted in the paper: Can use a reference-guided approach; this is of particular importance as all results presented appear to be from the reference-guided method.

Holley et al. focus their results on a correction comparison and show downstream results using an assembly comparison (using Flye) in the context of the human genome. For the correction comparison, they compare to FMLRC. In general, the results of Ratatosk seems to outperform FMLRC (with a larger run-time cost) on error correction. It also seems to create corrected reads that are better suited for variant calling, which is not too surprising given that FMLRC does not appear to have been specifically designed with that use case in mind. For assembly, they compare to results from a HiFi and HiCanu assembly as well as a recent Ash1 assembly. The results seem to be generally comparable to those assemblies with the best results in a few of the metrics.

Overall, the results are quite promising, especially in the context of diploid (e.g. human) genomes. The algorithmic/data structure usage is also of broad interest, and it seems like Ratatosk may further enable downstream analyses (compared to other hybrid correctors) that are currently still challenging. Additionally, the tool was downloaded and compiled following the instructions provided via GitHub (note: I did not run the method on any data).

However, the manuscript could benefit from some more details regarding the impact of the five features above, especially the reference-guided approach. Additionally, while reading the manuscript, I had a few questions around the process used to generate results that do not seem to be answered in the main document or an appendix/supplement.

Major Comments:

* Of the five features, the optional use of the reference-guided approach is potentially the most consequential, and simultaneously the least mentioned in the manuscript. This is of particular importance in the context of de novo assembly where a reference (even a low-quality reference) is frequently unavailable. While the method is designed to avoid overcorrection, the addition of a reference creates the possibility of reference bias in the results (note, I believe this is not very likely given the results from vcfeval). The authors also state that the reference guidance impacts both the correction and the computation time in the methods. Given that this approach is optional in Ratatosk, it seems appropriate that the results from both the reference-guided approach and the reference-free approach should be included in this document. Assuming there is an impact on the results (including downstream assemblies) and computation time, it should be discussed as well. To some readers, this may also present a more realistic or "fair" comparison to tools that did not assume the presence of a reference, both for correction and downstream assembly.

*Author' answer: We agree with the reviewer's comment that the reference-guided option favors Ratatosk in our experiments while FMLRC is purely reference-free. However, the impact on the results is most likely minor while the major benefit from this preprocessing is the running time. Indeed, previous hybrid correction tools have been evaluated for smaller genomes than human but Ratatosk was designed to be used on human genome data sets. While FMLRC is faster to run than Ratatosk in terms of CPU hours, both tools take a few weeks of real time computation to correct a single human genome. This running time is not sustainable for production use but the reference-guided approach makes it possible by scaling the Ratatosk correction to a network of compute nodes. Hence, rerunning Ratatosk without the reference-guided approach on the 18 data sets from our experiments, in addition to the downstream analysis and the subsampling experiment required by Reviewer 1, would add a significant delay to this review in addition to the computational resources required. This evidently highlights a current limitation of Ratatosk that we mention in the Conclusion. We also emphasize in the Results section that FMLRC is a reference-free approach.*

* Of the other four features, feature 4 has been discussed in previous papers (FMLRC at least, possible others; Wang et al., Table 1), but to my knowledge 1-3 have not. Feature 2 seems to be generally applicable to k-mer based correction, whereas features 1 and 3 seem to be more specific to ccdBG. It would be beneficial to discuss the impact that 1-3 each had on the corrected reads to give the reader a greater understanding of how much benefit comes from using the ccdBG data structure (i.e features 1 and 3) v. using a better method during read anchoring/correction (i.e. feature 2). Providing data/evidence to support this discussion would be ideal (as previous works have done with feature 4), but these features do not appear to be easily disabled by command-line parameters in Ratatosk.

*Author' answer: Feature 4 presented in Ratatosk is an extension of the two correction pass procedure from FMLRC. Both tools increase their k-mer length on the second round of correction. However, Ratatosk additionally colors the graph with the corrected long reads rather than with the input short reads during the second round of correction which improves the results. The novelty of feature 2 is the inexact k-mer anchoring, sometimes referred to as fuzzy match in the literature, which is not present in FMLRC nor other correction tools. Unfortunately, it is not possible to disable these features in Ratatosk.*

* The datasets used for testing use a mix a guppy v3.3 and v3.6, but most were generated with v3.3. The authors state that v3.6 offers better base-level accuracy. Presumably, the raw data for the Icelandic trios is available to the authors. If this is the case, why is mostly v3.3 data used? It adds a layer of complexity to the manuscript that seems to be avoidable.

*Author' answer: We agree that using two different Guppy versions adds a layer of complexity to the manuscript but it also brings the key advantage of showing the performance of Ratatosk and FMLRC on more erroneous data sets in addition to the PacBio data set which has the highest error rate. Because Guppy 3.6 is still a recent release, many ONT data sets stored in public repositories were basecalled with Guppy 3.3.*

* The authors suggest that "FMLRC is prone to overcorrection" after presenting the comparison results, but they don't seem to perform an equivalent comparison in terms of parameter selection (where available, of course). For example, Ratatosk appears to use a lower minimum threshold on "solid" (Tmin=2 or 1, depending on the pass) than FMLRC (assuming default, m=5). Given the sample coverage and the use case, the setting from Ratatosk is likely more appropriate (FMLRC appears to have been tested on non-human data with much higher coverage), but it is unclear how much of an impact that would have on the perceived overcorrection. Theoretically, the higher threshold on FMLRC would reduce the number of "solid" paths available for correction, so some of the over-corrections could just be a result of poor parameter choice for this use case. Additionally, the k-mer sizes in the two passes appear to be different with Ratatosk using longer k-sizes overall. The longer k-sizes are likely of benefit to Ratatosk given the inexact matching scheme, but may hinder FMLRC since it only uses exact solid matches. A more equivalent comparison would be ideal (i.e. identical or closer thresholds and k-sizes), but running that experiment is likely computationally expensive.

Instead, I suggest the authors specifically point out that these similar parameters are set differently and discuss how that may impact the comparison. If there are other comparable parameters aside from solid threshold and k-sizes, they should be included as well. Additionally, it would be beneficial to explain their choice of parameters, especially given that many other correction algorithms (including those not tested here) use a smaller k-size by default (17, 19, and 21 seem to be relatively common). Any evidence to support their parameter choices would also be beneficial in a supplement/appendix, even if only tuned using a subset of their data.

*Author' answer: Performing an equivalent comparison in terms of parameter selection is a difficult task as both tools have similar methods (k-mer based, two correction passes) but Ratatosk introduces several additional features. For example, Ratatosk relies heavily on the inexact k-mer anchoring which has no equivalent in FMLRC. Additionally, Ratatosk uses a very low minimum k-mer coverage to accommodate a wide range of input data sets while FMLRC might still use a higher minimum coverage (the paper mentions 5 but it is unclear if this threshold is still the one in use). This threshold is not a parameter of either methods. As suggested by the reviewer, we discuss these thresholds in the Results section.*

* For the correction analysis, what were the file types used for upstream alignment? Ratatosk seems to be capable of producing FASTQ files with quality scores, which has been noted to greatly benefit alignment and variant calling steps downstream. It's unclear what was used for Raw, while FMLRC only seems to produce FASTA files. Which file types were used should be stated clearly for the reader. If the FASTQ files were used in Ratatosk, it should be highlighted as a (likely) benefit of using Ratatosk over FMLRC and other tools that do not produce quality scores, and also noted as something likely potentially hindering the aligner/caller on FMLRC results.

*Author' answer: Ratatosk can indeed produce FASTA or FASTQ while FMLRC only outputs FASTA. In the case of a FASTQ output by Ratatosk, the output base quality scores are a direct representation of the result of Equation 2, i.e the confidence of Ratatosk in the correction of each base. However, none of the tools used in the downstream analysis actually takes advantage of base quality scores from the FASTQ files so results are identical in FASTA and FASTQ format. Quality scores mentioned in the manuscript are the ones from the VCF files output by Clair and they represent the quality of the variant call.*

* For the assembly comparison, how was Quast run? I assume a reference was used, and presumably it was hg38 based on the reference used during alignment. These should be stated.

*Author' answer: The Appendix of the revised manuscript includes the command lines used to run each tool for the evaluation. Furthermore, the Results section now mentions that all tools requiring a reference genome used the GRCh38.p13 human genome reference.*

* (I'm assuming the answer to the above comment is that it was compared to hg38) When reviewing the assembly results, I found it odd that the indels per 100kbp are all roughly the same while the mismatches per 100kbp are much lower from the Ratatosk/Flye assembly than either of the other two assemblies, especially given that Ash1 includes hg38 patches and that Ratatosk is designed to avoid overcorrection (i.e. preserve variation). Is there some justification for this discrepancy, or do the authors believe the entire difference is from error correction? If from correction, why is it not impacting indels the same way? Is this possibly a reference bias injected from reference-guidance? (This last question could be answered in part with reference-free results)

*Author' answer: The assembly evaluation now includes the assembly of the raw ONT reads with Flye. While the number of indels per 100 kbp in the raw assembly is about 4 times more than in any other assembly evaluated, the number of mismatches per 100 kbp is the second lowest after the assembly of the Ratatosk corrected ONT reads. This shows that the low rate of mismatches in the Ratatosk/Flye assembly is consistent with the rate of mismatches in the assembly of the raw data. It also discards the possibility of a reference bias as the assembly of the raw ONT reads is purely de novo. Finally, the low rate of mismatches in the raw ONT reads assembly is supported by the results from the FDA Precision challenge v2 (https://precision.fda.gov/challenges/truth/results) which shows that SNP calls obtained from raw ONT reads are now nearly as accurate as SNP calls obtained from Illumina reads.*

Minor Comments:

* I was unable to locate any settings used for FMLRC, Clair, Flye, Quast, or Merqury (versions are in the Appendix). These should be stated or "default" if no parameters were changed. Preferably, the exact commands for all steps of processing/analysis would be available in an appendix/supplement. Additionally, default parameters for Ratatosk are stated in the appendix. Were these the ones used to generate all results?

*Author' answer: The Appendix has been updated to include most command lines for each tool used in the manuscript. None of the default parameters of Ratatosk in the Appendix have been changed to run the experiments of the manuscript.*

* For table 3a, it would be beneficial to know what the selected "optimal" quality score is for each method (assuming they are different).

*Author' answer: The optimal quality scores thresholds are now provided in the Appendix for HG002, HG003 and HG004.*

* The GIAB version should be stated in the main text (it appears to be 3.3.2 from the Appendix).

*Author' answer: The revised manuscript indicates which version of the GIAB truth set was used. In addition, the results have been updated with the version 4.2 of the GIAB truth set (previously version 3.3.2).*

* The pre-processing requirements (CPU/memory) for Ratatosk should be listed separately under the Time and Memory section (even if comparably negligible) for completion. If available, it would likely be beneficial for FMLRC requirements as well for comparison (again, even if comparably negligible).

*Author' answer: For completeness, we listed in the Appendix of the revised manuscript the pre-processing requirements of Ratatosk and FMLRC for the HG002 ONT data set.*

* If the Icelandic trio data is publicly available (it may not be), it should be listed in Appendix.

*Author' answer: Access to the raw Icelandic sequence data is available on request from Kári Stefánsson at the premises of deCODE genetics. The data are not publicly available because of Icelandic state law. This is now mentioned in the Data section of the revised manuscript. However, all of our experiments are performed and are fully reproducible using the Ashkenazim trio from Genome In A Bottle.*

Suggestions:

* The authors may want to consider greater emphasis on the fact that their tests show Ratatosk working well on human datasets. Both reviews mentioned (Fu et al. and Zhang et al.) focused their benchmarks on relatively small non-human organisms (E. coli, yeast, etc), and neither seems to directly assess the impact on variant calling. To my knowledge, the tools evaluated in those benchmarks do not directly address these problems either (most were focused on de novo assembly, and preserving variation was likely secondary if considered at all). This would allow the authors to emphasize how the Ratatosk features address complexity of larger genomes, especially where preserving variation matters.

*Author' answer: We thank the reviewer for this suggestion. The revised manuscript now highlights the scaling to human genome data sets and the variant calling evaluation in the Abstract, Introduction and Conclusion.*

**Second round of review**

**Reviewer 1**

The authors' have addressed most of my concerns. I do still have one concern remaining regarding one comment:

My original comment: It says that a model was trained with Clair on HG002-1 dataset, but then the same model was used by Clair to call reads on the same dataset for the purposes of evaluation. Unless I am misunderstanding what was done, this would be equivalent to using the same training set as the validation set, which would allow Clair to overfit the data. This makes the result of Table 3 hard to interpret and leaves open the question of the SNP/Indel calling accuracy that would be achieved by Ratatosk and FMLRC in practice. I suggest that the authors address this in the text by using a training dataset that is different from the evaluation one. If there are no other datasets that can be used to achieve this, why not train the model on one chromosome and to the validation on the other chromosomes? (because the read set is human, reads could be pre-filtered by mapping according to chromosomes, so this should be feasible.)

Author' answer: In the revised manuscript, a model was trained independently for HG002, HG003 and HG004 for each raw and corrected data set. Subsequently, small variants of HG002 and HG004 were called with the model trained on HG003 while the small variants of HG004 were called with the model trained on HG002.

My new comment: I think that the new way of doing things is an improvement and the validation set is no longer the same as the training set. However, I still have concerns because the the set of variants in HG002 (son) is correlated with that of the parents (HG003 and HG004). Hence, the problem of overfitting may still exist when, e.g. running on HG002 after training on HG003. I am encouraged by the fact HG004 (father) results, using a model trained on HG003 (mother, unrelated), still give good results. Hence, I am OK with the experiments being the way they are; however, I do think a discussion is needed on how relatedness of individuals may affect the results in Table 4.

**Reviewer 2**

Most of the major comments from the first review are sufficiently addressed or discussed in the revised manuscript.

I do wish there was more data regarding the benefits of the five algorithmic features mentioned in my first review, as I believe this would be of great benefit to the community. However, I recognize that this is not a reasonable request given the current run-time of the method.

**Authors Response**

**Point-by-point responses to the reviewers’ comments:**

Reviewer 1 : I think that the new way of doing things is an improvement and the validation set is no longer the same as the training set. However, I still have concerns because the the set of variants in HG002 (son) is correlated with that of the parents (HG003 and HG004). Hence, the problem of overfitting may still exist when, e.g. running on HG002 after training on HG003. I am encouraged by the fact HG004 (father) results, using a model trained on HG003 (mother, unrelated), still give good results. Hence, I am OK with the experiments being the way they are; however, I do think a discussion is needed on how relatedness of individuals may affect the results in Table 4.

*Author's answer: We thank the reviewer for this suggestion. A note has been added in the main text about the risk of over-fitting because the individuals are related.*
